# Association of *mprF* mutations with cross-resistance to daptomycin and vancomycin in methicillin-resistant *Staphylococcus aureus* (MRSA)

**DOI:** 10.1038/s41598-020-73108-x

**Published:** 2020-09-30

**Authors:** Kanate Thitiananpakorn, Yoshifumi Aiba, Xin-Ee Tan, Shinya Watanabe, Kotaro Kiga, Yusuke Sato’o, Tanit Boonsiri, Feng-Yu Li, Teppei Sasahara, Yusuke Taki, Aa Haeruman Azam, Yuancheng Zhang, Longzhu Cui

**Affiliations:** Division of Bacteriology, Department of Infection and Immunity, Faculty of Medicine, Jichi Medical University, 3311-1, Yakushiji, Shimotsuke-shi, Tochigi, 329-0498 Japan

**Keywords:** Genetics, Microbiology, Pathogenesis

## Abstract

We first reported a phenomenon of cross-resistance to vancomycin (VCM) and daptomycin (DAP) in methicillin-resistant *Staphylococcus aureus* (MRSA) in 2006, but mechanisms underlying the cross-resistance remain incompletely understood. Here, we present a follow-up study aimed to investigate genetic determinants associated with the cross-resistance. Using 12 sets of paired DAP susceptible (DAP^S^) and DAP non-susceptible (DAP^R^) MRSA isolates from 12 patients who had DAP therapy, we (i) assessed susceptibility to DAP and VCM, (ii) compared whole-genome sequences, (iii) identified mutations associated with cross-resistance to DAP and VCM, and (iv) investigated the impact of altered gene expression and metabolic pathway relevant to the cross-resistance. We found that all 12 DAP^R^ strains exhibiting cross-resistance to DAP and VCM carried mutations in *mprF*, while one DAP^R^ strain with reduced susceptibility to only DAP carried a *lacF* mutation. On the other hand, among the 32 vancomycin-intermediate *S. aureus* (VISA) strains isolated from patients treated with VCM, five out of the 18 strains showing cross-resistance to DAP and VCM carried a *mprF* mutation, while 14 strains resistant to only VCM had no *mprF* mutation. Moreover, substitution of *mprF* in a DAP^S^ strain with mutated *mprF* resulted in cross-resistance and vice versa. The elevated lysyl-phosphatidylglycerol (L-PG) production, increased positive bacterial surface charges and activated cell wall (CW) synthetic pathways were commonly found in both clinical isolates and laboratory-developed mutants that carry *mprF* mutations. We conclude that *mprF* mutation is responsible for the cross-resistance of MRSA to DAP and VCM, and treatment with DAP is more likely to select for *mprF*-mediated cross-resistance than is with VCM.

## Introduction

Methicillin-resistant *Staphylococcus aureus* (MRSA) infections are serious clinical problems causing high morbidity and mortality worldwide. MRSA is resistant to not only β-lactam antibiotics but also other classes of antibiotics such as aminoglycosides, tetracyclines, or fluoroquinolones, restricting the available antibacterial agents for MRSA treatment^1–4^. Vancomycin (VCM), a glycopeptide antibiotic exerting bactericidal activity by binding to D-ala-D-ala residues of peptidoglycan to inhibit bacterial cell wall (CW) synthesis, is the first-line antibiotic against MRSA infections^5^. Emergence of MRSA with reduced susceptibility to VCM therefore further limits the scarcely available treatment options^3,4,6–8^.

Daptomycin (DAP), a cyclic lipopeptide antibiotic with potent bactericidal activity, is frequently used as salvage therapy after VCM treatment failure^9^. In the presence of calcium, the anionic DAP molecule attains its active cationic peptide form, which will then insert its lipophilic tail into the negative-charged cell membrane (CM)^10,11^. The interaction between DAP and the CM causes potassium leakage and membrane depolarization that ultimately contribute to cell death^12^. This means that DAP and VCM differ in not only chemical structure but also in their bactericidal mechanism^7,13^. Nevertheless, MRSA strains with cross-resistance to DAP and VCM, which was first reported by our group in 2006^14^, have been frequently isolated from patients treated with either DAP or VCM^8,15,16^.

Multiple peptide resistance factor (MprF) is reported to mediate DAP non-susceptibility in *S. aureus*^17^. Mutation of *mprF* is associated with gain-of-function, in which lysinylation of phosphatidylglycerol (PG) is enhanced, thus increasing membrane lysyl-phosphatidylglycerol (L-PG) production^17,18^. This positively charged L-PG will then be translocated from the inner membrane to the outer leaflet of the CM by the flippase domain of the MprF protein, causing an increased net positive charge on the CM^19^. Eventually, the more positively charged CM surface may serve as a protective barrier against DAP binding^20,21^. However, this remains controversial since only some DAP non-susceptible (DAP^R^) strains displayed enhanced L-PG concentration in outer leaflet although most DAP^R^ strains carrying *mprF* mutation showed increased L-PG production^19,22–25^. Aside from changes in CM properties, increased thickness of the CW is also proposed to cause ineffective binding of DAP to the CM^14,26^. Some DAP^R^ strains are accompanied by an increased expression of genes involved in CW synthesis, such as *murAB* or *pbp2*, a response similar to those induced by VCM and the other CW-targeting agents^27,28^. As a salient feature of vancomycin-intermediate *S. aureus* (VISA), CW thickening was reported one of the contributing factors to VCM resistance in some DAP^R^ strains. In fact, mutations in either *walK*, encoding the sensor protein kinase of a two-component regulatory system, or *vraSR*, involved in cell envelope homeostasis, both of which resulted in CW thickening, were related to the DAP/VCM cross-resistance^29,30^. However, phenotypic changes in CW thickness were not consistently observed in all DAP^R^ strains^26,31^. Consequently, the mechanism(s) conferring resistance of *S*. *aureus* to the two different classes of antibacterial agents remain largely unknown.

This study aimed to investigate genetic determinants of the cross-reduced susceptibility to DAP and VCM in clinically isolated MRSA. A total of 12 sets of DAP susceptible (DAP^S^) and DAP^R^ MRSA isolates collected from different hospitals in Japan were whole-genome sequenced, and gene mutations associated with the phenotype of cross-reduced susceptibility were identified and functionally characterized. Our results indicated that reduced susceptibility to both DAP and VCM was regulated by *mprF* mutation via increased L-PG production, subsequent alteration of bacterial surface charge, and CW biosynthetic pathways.

## Results

### Reassessment of VCM and DAP susceptibilities

This study began with analysis and validation of VCM and DAP susceptibilities for all paired isolates collected from 12 patients from whom DAP^R^ MRSA strains were generated during DAP therapy (Supplemental Table 1). DAP and VCM susceptibility tests on the 13 DAP^R^ strains could classify the DAP^R^ strains into two different resistance groups, judged by minimum inhibitory concentrations (MICs) and population analysis profiles, namely, cross-reduced susceptibility to DAP and VCM (termed reduced DAP/VCM susceptibility) and reduced susceptibility to only DAP (Table 1). Among the 13 DAP^R^ strains, 12 strains that belonged to the cross-reduced DAP/VCM susceptibility group showed DAP and VCM MICs of 1.5 to 3 mg/L. There is a 3.0- to 8.0-fold increase in DAP MIC and a 1.3- to 1.5-fold increase in VCM MIC of DAP^R^ strains when compared to their corresponding DAP^S^ parent strains, except for strain F-2 which showed an exceptional twofold increase in VCM MIC. One strain, K-2, showed reduced susceptibility to only DAP with a MIC of 2 mg/L but had no change in VCM susceptibility compared to its parent strain K-1 (Table 1). These susceptibility patterns were also confirmed by analysis of a resistant subpopulation against DAP and VCM (Supplemental Fig. 1) and determination of MICs with a different method (Supplemental Table 4). In addition, almost all DAP^R^ strains had increased doubling time compared to their DAP^S^ counterparts, but there were two exceptions (D-2 and G-2) (Table 1).

**Table 1:** Summary of MIC, gene mutation, MLST, doubling time, cell-wall thickness, cytochrome *c* uptake and L-PG content on the isolates from DAP treatment patients.

Patient	Strain	DAP MIC		VCM MIC		MLST	Mutation in		Doubling time (min)	Cell wall thickness (nm)	Cytochrome *c* uptake (%)^g^	L-PG content (%)^h^
		mg/L	Ratio^a^	mg/L	Ratio^a^		*mprF*	Others				
**Cross-reduced susceptibility group (to DAP and VCM)**												
A	A-1	0.38	1.00	1.5	1.00	764	–^b^	–	26.6	23.18 ± 2.50	100	100
	A-2	1.5	3.95	2	1.33	764	T345I	–	27.0	24.40 ± 1.72	73.27 ± 6.79*	177.20 ± 18.47*
B	B-1	0.19	1.00	1.5	1.00	764	–	–	30.0	23.28 ± 2.73	100	100
	B-2	1.5	7.89	2	1.33	764	L776S	B1_1709 (N31_fs)^c^	30.7	22.41 ± 1.58	78.75 ± 6.83**	128.86 ± 10.58*
C	C-1	0.5	1.00	2	1.00	764	–	–	29.5	24.28 ± 2.00	100	100
	C-3	1.5	3.00	3	1.50	764	A475P	–	30.2	22.17 ± 1.86	71.45 ± 4.51**	147.52 ± 18.09*
	C-4	3	6.00	3	1.50	764	L459_H466 del	–	30.9	23.17 ± 2.32	70.65 ± 11.63*	140.48 ± 11.44*
D	D-1	0.25	1.00	1.5	1.00	1	–	–	32.8	20.60 ± 2.77	100	100
	D-2	2	8.00	2	1.33	1	L826F	ir-1, ir-2^d^	27.6	19.86 ± 1.80	52.37 ± 15.09*	144.14 ± 12.20*
E	E-1	0.25	1.00	1	1.00	764	–	–	32.6	25.30 ± 2.46	100	100
	E-2	1.5	6.00	1.5	1.50	764	L826F	–	36.2	23.64 ± 2.67	61.79 ± 3.30**	166.21 ± 24.86*
F	F-1	0.25	1.00	0.75	1.00	764	–	–	33.3	24.89 ± 2.39	100	100
	F-2	2	8.00	1.5	2.00	764	L826F	*agrA*(T210I), F1_0943(A363T)	36.5	25.86 ± 2.15	71.98 ± 4.81*	124.00 ± 12.49*
G	G-1	0.5	1.00	1	1.00	2809	–	–	32.2	30.02 ± 2.32	100	100
	G-2	1.5	3.00	1.5	1.50	2809	T345A	ir	31.4	29.72 ± 2.15	45.99 ± 2.14**	158.88 ± 26.37*
H	H-1	0.75	1.00	2	1.00	5	–	–	27.1	23.31 ± 1.84	100	100
	H-3	0.5	0.67	2	1.00	5	–	*hisF*(G207_del), H1_0704(C241Y)	25.9	ND^e^	92.05 ± 4.73	89.62 ± 7.58
	H-5	3	4.00	3	1.50	5	L291I	*hisF*(G207_del), H1_0704(C241Y)	26.6	22.92 ± 2.35	78.46 ± 0.77**	155.3 ± 13.74*
	H-5(mprF_H-1)	0.5	1.00	2	1.00	5	–	*hisF*(G207_del), H1_0704(C241Y)	ND	ND	100	100
	H-3(mprF_H-5)	3	6.00	3	1.50	5	L291I	*hisF*(G207_del), H1_0704(C241Y)	ND	ND	69.26 ± 7.81*	149.26 ± 21.91*
I	I-2	0.75	1.00	2	1.00	NT	–	–	27.0	22.11 ± 1.83	100	100
	I-3	3	4.00	3	1.50	NT	W424R	–	33.8	25.55 ± 2.92**	68.88 ± 5.90*	165.51 ± 12.12*
J	J-1	0.38	1.00	1.5	1.00	764	–	–	27.1	23.44 ± 1.37	100	100
	J-3	1.5	3.95	2	1.33	764	L341S	–	30.1	23.40 ± 1.63	73.32 ± 3.19**	119.66 ± 7.16*
L	L-1	0.25	1.00	1.5	1.00	380	–	–	26.5	22.12 ± 1.80	100	100
	L-2	2	8.00	2	1.33	380	S337L	L1_0548(T134I)	29.9	22.24 ± 1.29	69.99 ± 9.15*	116.96 ± 4.30*
**Reduced susceptibility to only DAP group**												
K	K-1	0.38	1	1.5	1	764	–	*lacF*(trunc^)f^	24.7	21.50 ± 1.82	100	100
	K-2	2	5.26	1.5	1.00	764	–	*lacF*(H41)	27.1	21.15 ± 1.75	122.16 ± 1.00*	55.01 ± 15.16**

a) MIC ratio of DAP^R^ strain to its parent DAP^S^ strain; b) no mutation; c) fs: frameshift; d) ir: intergenic region; e) not determined; f) truncated at position 42; g & h) relative values compared to corresponding parent strains (*p < 0.05; **p < 0.01).

**Figure 1:**
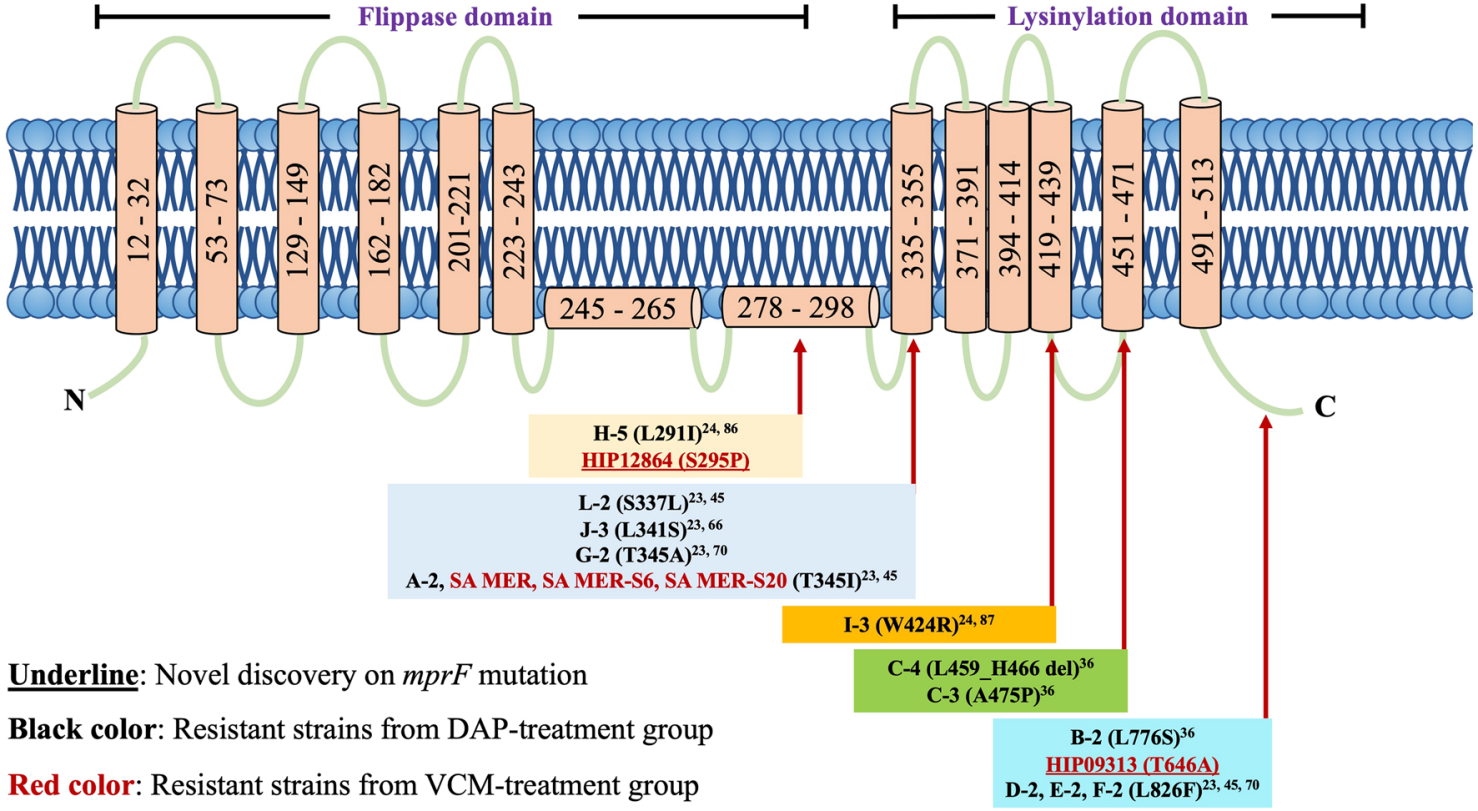
The location of *mprF* mutations in DAP^R^ strains and VISA strains. Most DAP^R^ (black text) and VISA (red text) isolates in this experiment carried *mprF* mutations on the lysinylation domain. The underlined mutations refer to newly discovered *mprF* mutations. The MprF structure is modified from previous studies^21^.

### Comprehensive mutation identification

To determine genomic alterations associated with reduced susceptibilities to DAP and VCM, whole-genome sequences of 12 pairs of DAP^S^ and DAP^R^ MRSA strains from 12 patients were determined. Comparative genome analysis found that all DAP^R^ strains with reduced susceptibility to both DAP and VCM, carried at least one non-synonymous mutation. All identified mutations were validated using PCR-based sequencing and are listed in Table 1. Interestingly, these strains unanimously carried mutations on *mprF* gene encoding an L-PG synthetase, which is known to synthesize positively charged lipid L-PG. On the other hand, the DAP^R^ strain K-2 with reduced susceptibility to only DAP had an insertion mutation in *lacF*, which encodes a conserved ATP-binding domain homologous to ABC transporters known in bacteriocin immunity systems^32^. The *lacF* of K-1 differed from that of K-2 for the presence of one thymine deletion at position 125 that generated a premature stop codon (Supplemental Fig. 2A) and resulted in LacF truncation at position 42 (Supplemental Fig. 2B), indicating that the restoration of LacF function is responsible for reduced susceptibility of K-2 to DAP.

**Figure 2:**
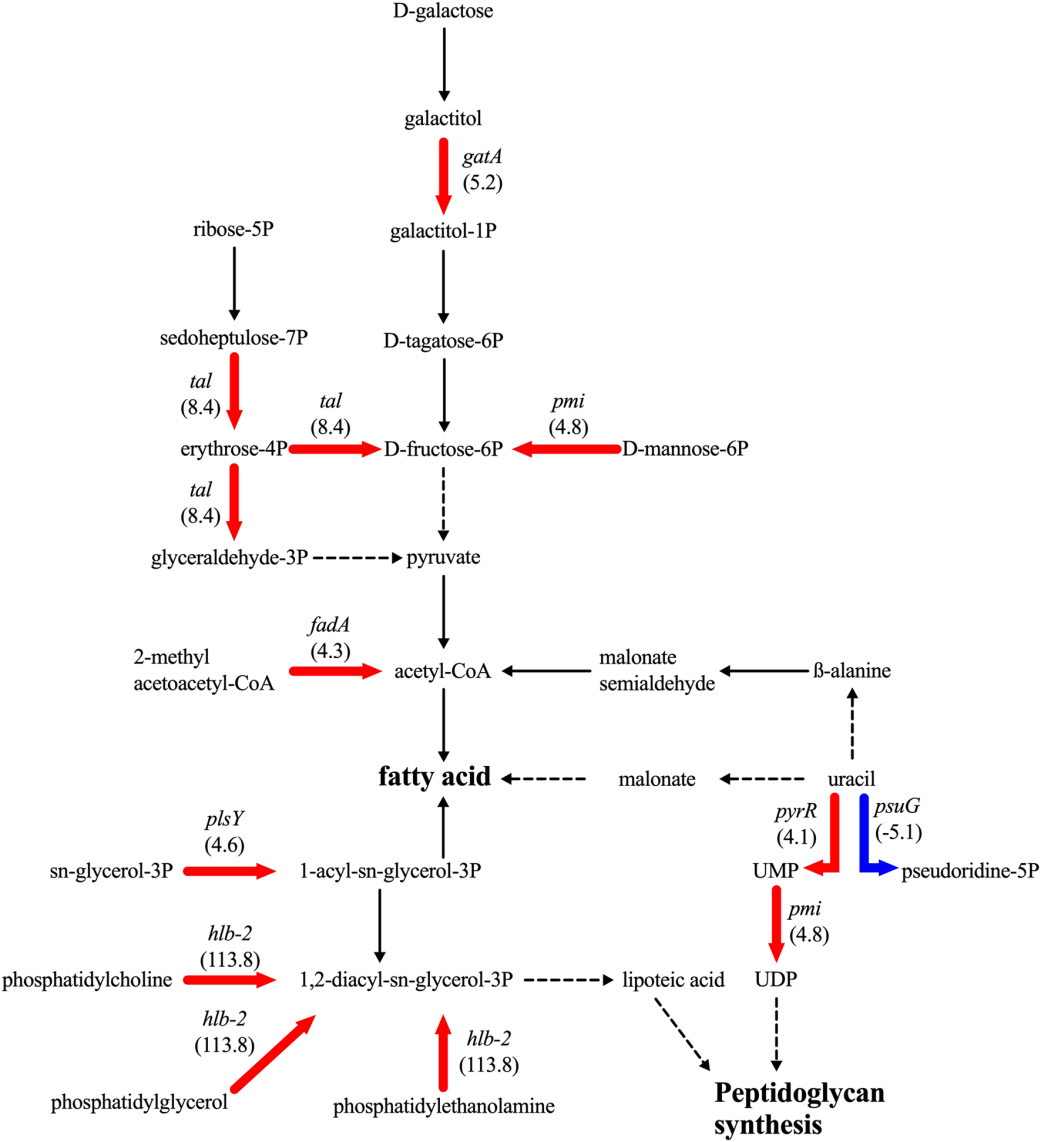
Gene expression in contribution to cross-reduced susceptibility in DAP^R^ strain H-5. Increased gene expression in fatty acid and peptidoglycan via carbohydrate metabolism (galactitol, ribose, or mannose) was observed. The red arrows refer to gene upregulation. The blue arrows refer to gene downregulation.

For the *mprF* mutation, 10 types of point mutations were identified in this study, most of which were located on the lysinylation domain of MprF (Fig. 1). In addition, as shown in Table 1, DAP^R^ strains D-2 and G-2 carried intergenic region mutations besides the *mprF* mutation. Four out of 11 cross-reduced susceptibility strains had additional mutations that resulted in amino acid substitutions of B1_1709(N31_fs) in DAP^R^ strain B-2, *agrA*(T210I) and F1_0943(A363T) in DAP^R^ strain F-2, L1_0548(T134I) in DAP^R^ strain L-2, and *hisF*(G207_del) and H1_0704(C241Y) in DAP^R^ strains H-5. The mutations of *hisF*(G207_del) and H1_0704(C241Y) could also be found in DAP^S^ strain H-3, indicating that these mutations seem to not be directly involved in the mechanism of reduced DAP/VCM susceptibility. In summary, *mprF* mutations were commonly found in the MRSA isolates with cross-reduced susceptibility to DAP and VCM that were isolated from patients who had DAP therapy.

### Detection of genes reported to be associated with decreased susceptibility to VCM or DAP in *S. aureus*

Many genes have been reported to be associated with conversion of vancomycin-susceptible *S*. *aureus* to VISA, including *walK*, *clpP*, *graSR*, *vraSR*, *msrR*, and *rpoB*^30,33,34^. Some of these VISA-related genes were also reported to reduce DAP susceptibility in MRSA^33,35^. Therefore, we examined the sequences of these genes for all strains in DAP treatment group, but no differences were found between any pair of DAP^S^ and DAP^R^ strains. The phenomenon of DAP and VCM cross-resistance was first reported in VISA strains in 2006^14^, and afterward, it became recognized in clinical settings during the VCM therapy. Therefore, we further investigated and characterized the pattern of DAP and VCM susceptibilities for 32 VISA strains isolated from patients worldwide during years of 1996 – 2004 when the DAP was not available in clinical setting^34^. We found that among the 32 VISA strains, 18 showed both intermediate VCM resistance and reduced susceptibility to DAP (cross-reduced susceptibility) (Table 2). All 32 strains were examined for single nucleotide polymorphisms (SNPs) associated with VCM resistance (Table 2). We also determined full *mprF* sequences for all 32 VISA strains using PCR and Sanger sequencing methods, and the results were combined with our previous results on the SNPs of *walK*, *clpP*, *graSR*, *vraSR*, *msrR*, and *rpoB* in Table 2. We found point mutations in *mprF* in five out of 18 VISA strains with cross-reduced susceptibility to DAP and VCM, but not in any of the 14 strains with only intermediate VCM resistance. No other mutations found in genes or intergenic region of cross-reduced susceptible DAP^R^ strains from DAP-treated patients were identified in the VISA isolates. These results suggested that there is a high prevalence of reduced DAP/VCM susceptibility among the VISA strains and that *mprF* mutations play a role in conferring the cross-reduced susceptibility to VCM and DAP in some VISA strains that were generated during the prolonged VCM chemotherapy.

**Table 2:** Summary of MIC and gene mutation of VISA strains.

Strain name	Etest MIC (mg/L)		Gene Mutations^a^								
	DAP	VCM	*mprF*	*walK*	*clpP*	*graS*	*graR*	*vraS*	*vraR*	*msrR*	*rpoB*
**Cross-reduced susceptibility group (to DAP and VCM)**											
MI (HIP5827)	1.5	6	–	V494L^30^	–	–	–	–	–	–	R140S^34^
SA MER	2	3	T345I	–	ND	ND	ND	ND	ND	ND	ND
SA MER-S6	4	3	T345I	–	ND	ND	–	–	ND	–	ND
SA MER-S20	4	6	T345I	–	ND	ND	–	–	ND	–	–
HIP06297 (PC)	2	4	–	A567D^30^	ND	ND	–	–	ND	–	Q468L^34^
HIP08926	2	3	–	R222I, T492K^30^	ND	L26F, I59L, T224I^30^	D148Q^30^	–	ND	–	ND
HIP09143	1.5	3	–	–	ND	ND	–	–	ND	–	ND
HIP12864	2	4	S295P	–	ND	ND	–	–	ND	–	P519L^34^
HIP13057	1.5	4	–	R282C^30^	ND	ND	E15K^30^	–	ND	–	H481Y^34^
HIP13036	1.5	6	–	–	ND	ND	–	T104A^26^	ND	–	–
Mu50	1.5	6	–	–	–	–	N197S^30^	I5N^26^	–	E146K^34^	H481Y^34^
HIP06854	1.5	6	–	T492K^30^	ND	ND	ND	ND	ND	ND	ND
HIP09313	2	4	T646A	L10F, S437T^30^	R152H^26^	ND	ND	P327S^26^	E59D^30^	ND	ND
HIP09662	1.5	3	–	Ins 433N, Ins 434D^30^	ND	L26F, I59L, T224I^30^	D148Q^30^	–	E59D^30^	–	D471N, S486L^34^
LY-1999–01	1.5	4	–	N48K, R222K, A468T^30^	ND	L26F, I59L, T224I^30^	D148Q^30^	ND	E59D^30^	K321R^34^	R406S^34^
99/3700-W	1.5	3	–	R222K, V366M, A468T^30^	ND	L26F, I59L, T224I^30^	D148Q^30^	–	–	–	–
28,160	1.5	3	–	ND	ND	L26F, I59L, T224I^30^	D148Q^30^	ND	E59D^30^	ND	S529L^34^
BR5	1.5	3	–	R222K, V366M, A468T^30^	ND	L26F, I59L, T224I^30^	D148Q^30^	–	E59D^30^	ND	I527M^30^
**Intermediate resistance to only VCM group**											
NJ (HIP5836)	0.75	4	–	I28T, 1341V^30^	–	–	S79F^30^	A260V^30^	–	–	H481Y^34^
HIP07256	1	3	–	ND	ND	ND	ND	ND	ND	ND	ND
LIM2	1	3	–	ND	ND	L26F, I59L, T224I^30^	D148Q^30^	–	E59D^30^	ND	H481N, S529L^34^
HIP09740	0.75	3	–	V380I^30^	ND	ND	–	–	ND	–	H481D^34^
BR15	1	3	–	ND	ND	ND	ND	ND	ND	ND	ND
HIP10540	1	4	–	–	ND	L26F, I59L, T224I^30^	D148Q^30^	–	ND	–	A477V^34^
P1V44	0.75	3	–	–	ND	L26F, I59L, T224I^30^	D148Q^30^	–	ND	–	H481N, S529L^34^
99/3759-V	0.75	3	–	V156Q^30^	M1V^26^	L26F, I59L, T224I^30^	D148Q^30^	ND	E59D, H481N, S539L^30^	ND	H481N, S529L^34^
AMC11094	0.75	3	–	ND	ND	ND	–	–	A113V^30^	–	–
LY-1999–03	1	4	–	N48K, R222K, A468T^30^	ND	L26F, I59L, T224I^30^	D148Q^30^	–	E59D^30^	K312R^30^	–
C2000001227	1	4	–	A243T^30^	ND	ND	–	A314V^30^	ND	–	–
NRS118	1	4	–	F330S^30^	ND	ND	–	–	ND	–	H481N, S529L^34^
NRS126	0.5	3	–	–	ND	ND	–	–	ND	–	H481N^34^
98,141	0.75	3	–	–	ND	L26F, I59L, T224I^30^	D148Q^30^	ND	E59D^30^	ND	H481N, S529L^34^

a) *mprF* mutation was determined in this study, and the other gene mutations were detected in the previous studies (references were indicated); -: no mutation; ND: Not determined.

### Substitution of *mprF* in DAP^S^ strain with mutated *mprF* identified in the DAP^R^ strain caused reduced DAP and VCM susceptibility

To confirm the role of *mprF* mutations in reduced DAP and VCM susceptibility, an identified *mprF* mutation (*mprF*(L291I)) of DAP^R^ strain H-5 was cloned and introduced into its corresponding DAP^S^ strain H-3 to replace *mprF* of H-3. The H-3 strain (an isogenic strain of H-1 and H-5), isolated in between H-1 and H-5 during DAP therapy, was chosen for *mprF* substitution to eliminate the confounding effect of other gene mutations (*hisF* and H1_0704), which cannot be found in DAP^S^ strain H-1 (Table 1). The MIC test showed that the H-3 strain carrying *mprF*(L291I) had increased MICs of both DAP and VCM, from 0.5 and 2 respectively, to 3 mg/L, showing reduced DAP/VCM susceptibility to the same levels of the H-5 strain; vice versa, replacement of *mprF*(L291I) of H-5 with *mprF* of H-3 resulted in decreased MICs of both DAP and VCM from 3 to 0.5 and 2 mg/L, respectively, for the H-5 strain (Table 1). These results demonstrated that *mprF* mutations cause reduced susceptibility to both DAP and VCM in the MRSA H-5 strain.

### Reduced DAP and VCM susceptibility associated with *mprF* mutation was found in in vitro selected mutants

The clinical DAP^R^ strains isolated from patients who had DAP therapy exhibited reduced susceptibility to both DAP and VCM due to *mprF* mutations. The mutation position in *mprF* varied among strains from different patients, as shown in the above results (Table 1, Fig. 1) and the findings of Kanesaka et al.^36^. To understand whether this is also the case for in vitro selected DAP^R^ mutants with cross-reduced DAP/VCM susceptibilities, we generated DAP^R^ strains with reduced DAP/VCM susceptibility in vitro by exposing DAP^S^ MRSA to DAP of gradually increasing concentrations and examined mutations for *mprF* and *lacF*. Two DAP^R^ mutants were obtained from DAP^S^ strain C-1 (DAP MIC, 0.5 mg/L) by stepwise selection on Mueller Hinton (MH) agar containing increasing DAP concentrations from 0.5 to 4 mg/L. These mutants could grow in the presence of 4 mg/L DAP. We found that these two mutants had reduced susceptibility to both DAP and VCM, increasing the MICs of DAP from 0.5 to 3 and 6 mg/L and VCM from 2 to 3 and 4 mg/L, and are accompanied by *mprF* mutations, *mprF*(T472K) for the mutant C-1_DAP^R^#1 and *mprF*(R50L) for mutant C-1_DAP^R^#2, respectively (Table 3).

**Table 3:** Summary of MIC, doubling time and mutations in *mprF* and *lacF* on in vitro derivatives of the C-1 and K-1 strains.

Strain	Etest MIC (mg/L)		Doubling Time (min)	Mutation in	
	DAP	VCM		*mprF*	*lacF*
**Clinical isolates from Patient C**					
C-1	0.5	2	29.5	–	–
C-3	1.5	3	30.2	A475P	–
C-4	3	3	30.9	L459_H466 del	–
**In vitro derivatives of C-1 strain**					
C-1_DAP^R^#1	3	3	30.8	T472K	–
C-1_DAP^R^#2	6	4	28.0	R50L	–
**Clinical isolates from Patient K**					
K-1	0.38	1.5	24.7	–	trunc*
K-2	2	1.5	27.1	-	H41
**In vitro derivatives of K-1 strain**					
K-1_DAP^R^#1	6	3	54.6	T472K	H41A
K-1_DAP^R^#2	6	3	35.4	T472K	H41A

–: no mutation; DAP^R^: Daptomycin non-susceptible strain; *: truncated at position 42.

The DAP^R^ strain K-2 with reduced susceptibility to only DAP carried a *lacF* mutation that has not been previously reported (Table 1). To valuate this mutation, a similar stepwise DAP selection was performed on its DAP^S^ counterpart strain K-1, and two DAP-resistant mutants (K-1_DAP^R^#1 and K-1_DAP^R^#2) were generated. Interestingly, these two in vitro selected mutants showed decreased susceptibility to both DAP and VCM, increasing the MICs of DAP from 0.38 to 6 mg/L and VCM from 1.5 to 3 mg/L; it was also accompanied by a *mprF* mutation in addition to the restoration of LacF as seen in K-2 strain (Table 3 and Supplemental Fig. 2B). These results, together with the above study on clinical DAP^R^ strains, demonstrated that the phenomenon of reduced susceptibility to DAP and VCM in MRSA is strongly associated with *mprF* mutations.

### Reduced DAP/VCM susceptibilities and CW thickness

Thickened CW is known as a phenotypic determinant for VCM resistance in VISA^7,8,37^. Although not consistently reported, alteration of CW structure and/or changes in expression of genes involved in CW metabolic pathways have also been found in some DAP^R^ strains^26,31^. Therefore, alteration of CW structure might be one of the factors involved in reduced DAP and VCM susceptibility. In order to test this hypothesis, the CW thickness of 30 cells from each DAP^S^/DAP^R^ strain were measured by transmission electron microscopy (TEM). The results showed that only one strain in the group of cross-reduced DAP and VCM susceptibility (I-3) carrying a *mprF* mutation (*mprF*(W424R)) displayed significantly increased CW thickness (25.55 ± 2.92 nm) compared with its susceptible counterpart I-2 (22.11 ± 1.83 nm) (Table 1). In contrast, the other 11 DAP^R^ strains in reduced DAP/VCM susceptibility group did not exhibit significant CW thickening compared with their corresponding parental strains (Table 1).

There was also no difference in CW thickness between DAP^S^ isolate K-1 (21.50 ± 1.82 nm) and DAP^R^ isolate K-2 (21.15 ± 1.75 nm) exhibiting resistance to only DAP due to a *lacF* mutation (Table 1). These results did not clearly support the association of CW thickening with the phenomenon of reduced susceptibility to DAP in the DAP^R^ strains with *mprF* or *lacF* mutations.

### *mprF* mutation and bacterial surface charge

The *mprF* mutation had been previously reported in MRSA with reduced DAP susceptibility. MprF is a membrane-bound enzyme that adds lysine to phosphatidylglycerol in the cytoplasmic membrane. This modification is reported to change the electrostatic repulsive forces of the bacterial CM, which then conferred reduced susceptibility to cationic antimicrobial peptides^19,23^. To determine whether *mprF* mutations identified in this study resulted in such alterations, we carried out a cytochrome *c* binding assay on all DAP^R^ strains to examine the alteration of bacterial surface charges. A cationic cytochrome *c* can bind a negatively-charged bacterial cell surface and, hence, has been widely employed to determine the relative surface charges of the cell envelope^38,39^. Our results showed that all strains carrying a *mprF* mutation had significantly reduced cytochrome *c* binding when compared to their parental strains, indicating that all *mprF* mutations identified in this study caused increased positive surface charge (Table 1). Similarly, increased positive charge on bacterial surface was observed when the *mprF* of DAP^S^ strain H-3 was replaced with mutated *mprF*, while replacement of mutated *mprF* in DAP^R^ strain H-5 with that of its wild-type counterpart reduced the positive surface charge (Table 1). The DAP^R^ strain with reduces susceptibility to only DAP carrying a *lacF* mutation did not exhibit reduced negative charges (Table 1). These results suggested that alteration of bacterial surface charge is associated with *mprF* mutation-mediated reduced DAP/VCM susceptibility in MRSA.

### *mprF* mutation and L-PG production

The increased cationic phospholipid L-PG production in cytoplasmic membranes has been reported to decrease DAP susceptibility in MRSA^40^. To understand whether the *mprF* mutations found in this study are implicated in L-PG production, we set out to determine membrane L-PG levels for all DAP^R^ and DAP^S^ strains using the thin-layer chromatography (TLC) assay. Altered L-PG production of DAP^R^ strains over corresponding DAP^S^ strains was calculated in relative values (percentages) and is summarized in Table 1. As shown in Table 1, all DAP^R^ strains with cross-reduced DAP/VCM susceptibility (carrying a *mprF* mutation) showed increased L-PG production. Although a more than 50% increase in L-PG production can be found in most DAP^R^ strains, four strains (B-2, F-2, J-3, and L-2) harboring *mprF* mutations at different locations displayed only a marginal increase (10% or 20%) (Table 1). These results suggested that increased L-PG production regulated by *mprF* mutation may contribute to cross-reduced DAP/VCM susceptibility. In addition, we found that the K-2 strain with reduced susceptibility to only DAP (carries *lacF* mutation) had decreased L-PG production compared to its DAP^S^ counterpart. This indicated that *lacF* mutation may raise reduced susceptibility to DAP through a different metabolic pathway from what has been reported so far.

### Transcriptional analysis on representative DAP^R^ strains carrying *mprF* or *lacF* mutation and their DAP^S^ counterparts

In the above results, the association of *mprF* mutation and altered membrane metabolic pathways with cross-reduced DAP/VCM susceptibility was clearly demonstrated; however, the impact of the *mprF* mutations found in this study on metabolic regulations toward the cross-reduced susceptibility remains to be clarified. To this end, a representative pair of DAP^S^ and DAP^R^ strains, H-3 and H-5, isolated from patient H were selected for a whole-genome-scale gene expression profiling by RNA-sequencing. The *mprF*(L291I) mutation identified in H-5 is the only genomic alteration found between H-5 and H-3 and is considered to be responsible for cross-reduced susceptibility to DAP and VCM, as verified by gene substitution experiments (see elsewhere above). A total of 103 genes differentially expressed by more than fourfold between H-3 and H-5 were found (Supplemental Table 5). Among them, 61 genes were upregulated (59.22%) and 42 genes were downregulated (40.78%). These genes could be roughly classified into four functional categories: metabolism (27.18%), information storage and processing (13.59%), cellular process and signaling (6.80%), and the others (52.43%). As shown in Supplemental Table 5, a number of genes directly or indirectly involved in metabolism of fatty acid and peptidoglycan are found to be upregulated in DAP^R^ strain H-5. These include genes responsible for fructose-6-phosphate (F-6P) synthesis such as *gatA* (5.2-fold), *tal* (8.4-fold), and *pmi* (4.8-fold); or fatty acid synthesis such as *hlb-2* (113.8-fold), *fadA* (4.3-fold) and *plsY* (4.6-fold), all of which were highlighted on the map of fatty acid metabolic pathway (Fig. 2). It was also noted that seven out of 61 upregulated genes in DAP^R^ strain H-5 were involved in CW metabolism. Upregulation of *nagD* (6.6-fold), *pyrR* (4.1-fold) and *pmi* (4.8-fold) genes and downregulation of *psuG* (-5.1-fold) collectively affect the intracellular pool of uridine diphosphate-*N*-acetylglucosamine (UDP-NAG) and UDP-*N*-aceylmuramic acid (UDP-NAM), which serves as backbone for peptidoglycan (Fig. 2)^41^. In addition, upregulation of the genes associated with staphylococcal “cell wall stimulon”^28,42,43^, such as *spsA* (4.3-fold), *ssaA* (5.9-fold), *relP* (5.8-fold) and *sasA* (4.3-fold) was also found. Thus, mechanism of reduced DAP/VCM susceptibility by *mprF* mutation may be resulted from changes in CW/CM metabolism.

Unlike the association between *mprF* mutations and reduced DAP/VCM susceptibility, which can be deduced from our current study, the regulatory function of a *lacF* mutation on DAP single resistance in the K-2 strain is still unclear, prompting us to perform RNA-Seq analysis. The gene expression profiles of K-1 and K-2 strains are shown in Supplemental Table 6. There are 37 (68.52%) upregulated and 17 (31.48%) downregulated genes with a fourfold change between the DAP^S^ (K-1) and DAP^R^ (K-2) strains. Fifty percent of the differentially expressed genes are involved in amino acid or carbohydrate transport and metabolism, and energy production and conversion. This is followed by 11.11% of genes associated with defense mechanism and 7.4% with CW, CM, and envelop metabolisms. Among those upregulated genes, six *lac* operon genes (*lacABCDEG*) that comprise tagatose 6-phosphate pathway and lactose- and galactose- metabolizing enzymes overexpressed by 18- to 74-fold. In addition, altered expression in genes associated with CM metabolism were identified, including upregulation of *acyP* (4.9-fold), *fabZ* (5.8-fold), and *metI* (9.6-fold), and downregulation of *lctE* (-14.8-fold). Alteration of cellular defense associated genes was also found in the K-2, such as overexpression of *gpxA2* (6.8-fold) and downregulation of *opp-4D* (-4.1-fold).

## Discussion

The current study was conducted to investigate genetic determinants of cross-reduced susceptibility to DAP and VCM in MRSA. DAP and VCM are two different classes of antibiotics exhibiting distinct modes of bactericidal actions, thus triggering different resistance mechanisms in bacterial strains. Nevertheless, MRSA strains with reduced susceptibility to both DAP and VCM, a phenomenon known as cross-resistance to DAP and VCM, have been reported^14–16^. Owing to the fact that DAP and VCM are primary treatment options for MRSA infections, understanding the regulatory pathways leading to cross-resistance is crucial to facilitate the identification of novel target sites and the development of new therapeutic agents, contributing to the management of difficult-to-treat bacterial infections.

MprF is known to play a role in protecting bacteria against cationic antimicrobial peptides (CAMPs), including DAP, by altering bacterial membrane surface charges. Principally, MprF regulates the transition of phospholipid PG to L-PG by the addition of a lysine residue, causing an increased positive charge in CM, which is repulsive toward cationic antibiotics^13,16,38^. Accordingly, cells lacking the *mprF* gene showed increased susceptibility toward many positively charged antibiotics, including CAMPs, DAP, or VCM^16,44^. Previous studies frequently attributed reduced DAP susceptibility to *mprF* mutation, but a few mentioned about its association with alteration of VCM susceptibility^25,45,46^. Our current study demonstrated that *mprF* mutations are major determinants of cross-reduced susceptibility to DAP and VCM in MRSA during DAP therapy but have only partial contribution during the course of VCM chemotherapy (Table 1 and 2). The cross-reduced susceptibility to DAP and VCM mediated via *mprF* mutation was confirmed by a gene replacement assay whereby introduction of *mprF* mutation from DAP^R^ strain H-5 to DAP^S^ strain H-3 resulted in increased DAP and VCM MICs of DAP^S^ strain H-3 (Table 1). This cross-reduced susceptibility by *mprF* mutation might partially be explained with the change of bacterial surface charges by increment of lysyl-PG (L-PG) in the mutant strains (see later). Moreover, in our study, we showed that *mprF* mutations contribute to increase in MICs of DAP and VCM located on the lysinylation domain (Fig. 1). The mutation in the lysinylation domain of *mprF* is not just limited to clinical isolates, since most laboratory-derived reduced DAP/VCM susceptibility isolates obtained by stepwise DAP selection on DAP^S^ strains from both cross-reduced susceptibility group (strain C-1) and single-reduced susceptibility group (strain K-1) also carried *mprF* mutations in the lysinylation domain (Table 3).

Despite having a pronounced association with DAP-selected cross-reduced susceptibility strains, involvement of *mprF* mutations in the reduced DAP/VCM susceptibility during VCM exposure is less evident. Regardless, glycopeptide-resistant bacterial isolates exhibiting reduced DAP/VCM susceptibility phenotype can be observed in previous^14,47^ and current studies (Table 2). VISA strains display thickened CW to allow increased binding of VCM to false targets in peptidoglycan (affinity trapping), thereby contributing to their reduced VCM susceptibility^8,14^. Similar to VCM, the target site of DAP is located in the CM. Moreover, DAP is bigger than VCM in molecular sizes (1,620.67 for DAP and 1,485.7 for VCM). DAP molecules need to penetrate through the CW, the primary barrier of bacterial defense mechanism, before reaching their lethal targets. Therefore, one possible pathway leading to DAP resistance in VISA strains may be increased CW thickness^7,37^. CW thickening could also explain the reduced DAP susceptibility in DAP-selected reduced DAP/VCM susceptible strains, as reduced DAP binding at CM was observed in DAP^R^ strains of *Enterococcus*^48,49^. However, as shown by our TEM analysis, only one DAP^R^ strain (I-3; Table 1) has thickened CW. Neither the remaining 11 sets of DAP^R^ strains from the group of reduced DAP/VCM susceptibility carrying *mprF* mutation nor DAP^R^ strain with reduced susceptibility to only DAP carrying *lacF* mutation showed increased thickness of CW (Table 1). Thus, increased cell wall thickness is not a common phenotype in clinical DAP^R^ strain with cross-reduced susceptibility.

Nonetheless, similar to previous observations^7,26,37^, changes in the expression of CW-related genes have been identified in both DAP^R^ and VISA strains with cross-reduced susceptibility to DAP and VCM. According to our RNA-Seq differential expression analysis, reduced DAP/VCM susceptibility seems to be associated with altered CW metabolism, although most DAP^R^ strains did not show significant differences in CW sizes compared with their parental strains. This contrasting phenomenon might due in part to the small range of VCM MIC changes observed between DAP^S^ and DAP^R^ strains. In addition, DAP/VCM cross-resistance has been reported in both laboratory-derived and clinical isolates with no phenotypic characteristic of CW thickening^31^. It is therefore indicated that DAP/VCM cross-resistance is not the result of only one contributing factor; while increased CW thickness is associated with DAP and VCM cross-resistant VISA strains, alteration in membrane surface changes is more likely the causative factor of DAP and VCM cross-resistance in DAP^R^ strains. This hypothesis can be supported by several previous studies that reported that not only CW alteration but also changes in CM properties could be a substantial factor leading to cross-reduced DAP/VCM susceptibility^11,12,39,50,51^.

Daptomycin is a lipopeptide antibiotic acting on bacterial CM^10,11^. Membrane depolarization and ion leakage can be observed when the positively charged Ca^2+^-DAP forms a complex with the negatively charged hydrophilic head group of PG and bactoprenol-bound cell wall precursor in CM^11,12,52^. Therefore, phenotypic alteration of membrane surface charges via *mprF* mutation is a commonly reported bacterial evolution to resist positively charged drugs, such as CAMPs and DAP^12,39,50,51^. Interestingly, VCM molecules contain an ionizable amine and carboxylic group, which also display positive charge when administered^53,54^. Moreover, disruption of negatively charged wall teichoic acids (WTA) by deletion of the *dltABCD* operon involved in alanylation of teichoic acids was reported to increase the drug susceptibility of *S*. *aureus* Sa113 to both CAMPs, such as α-defensins or nisin, and glycopeptides, such as VCM or teicoplanin^55,56^. Hence, we postulated that a change in net surface charge as mediated by *mprF* mutation seems to be able to confer reduced DAP and VCM susceptibility in bacterial strains.

Herein, every DAP^R^ isolate in the group of reduced DAP/VCM susceptibility carrying *mprF* mutations in different positions, as well as DAP^S^ strain H-3 transformed with *mprF* mutation, exhibited significant alteration of surface charge as implicated by reduction of cytochrome *c* binding in these strains compared with the DAP^S^ strains (Table 1). These observations attributed decreased DAP/ VCM susceptibility to reduction of negative cell surface charges. According to our results, this mechanism seems to be regulated by *mprF*, although *rpoB* mutations have also been reported to alter surface charges^35^. The change in bacterial membrane surface charges results from the modification of anionic PG to cationic L-PG by the lysinylation domain of MprF^57^. DAP^R^ isolates were indeed consistently reported to have increased L-PG production due to *mprF* mutations^23,39,58^. In concordance, our results showed that L-PG production in DAP^R^ isolates of the cross-reduced DAP/VCM susceptibility group increased in a mutation site-dependent pattern. The cause-effect relationship between increased L-PG production and *mprF* mutation is further confirmed in our study by transformation of mutated *mprF* in the DAP^S^ strain (Table 1). Moreover, RNA-Seq analysis showed changes in gene expression that enhance fatty acid biosynthesis (DAP^R^ strain H-5), which indirectly facilitate the production of L-PG. Among, upregulation of genes involved in generation of F-6P (*gatA*, *tal* and *pmi*) were observed (Supplemental Table 5, Fig. 2). The F-6P is the key substrate for pyruvate biosynthesis, a crucial metabolite of the citric acid cycle required for energy metabolism. The upregulation of this process inevitably increases the intracellular pool of acetyl-CoA^59–61^, which serves as a precursor for fatty acid biosynthesis catalyzed by acetyl-CoA carboxylase and many acyl-carrier protein^62^. We postulated that these differential gene expressions will favor alteration of membrane property, since enhanced fatty acid synthesis will facilitate CM biosynthesis and subsequently increase the supply of building block for L-PG production by using acetyl-CoA as a precursor. In support of our hypothesis, mutation in acetyl-CoA synthetase in combination with other mutations has been reported to contribute to DAP^R^^17^.

Although different locations of mutations in *mprF* have been proposed to affect L-PG production and consequently DAP susceptibility^23,25^, the levels of L-PG production varied even when host cells do not carry *mprF* mutations or carry the same *mprF* mutation site, as shown in the study by Mishra et al.^63^. Many reports also showed that DAP^R^ strains carrying *mprF* mutation exhibited increased intracellular L-PG production, but the ratio of outer leaflet L-PG is not differ from DAP^S^ strains^22,25,64–66^, possibly due to reduced intradomain interaction^25^. Therefore, whether or not increased L-PG production directly affect cross-reduced DAP/VCM susceptibility needs to be further investigated. Nevertheless, our results indicated that changes in surface charge, enhanced levels of L-PG production and alteration of CW metabolism, presumably regulated by *mprF*, can contribute to reduced susceptibility to both DAP and VCM.

The *mprF* mutation is not a unique genetic determinant correlated with cross-reduced susceptibility phenotype. Other mutations have been reported to be responsible for reduced DAP and VCM susceptibility in laboratory mutants (RNA polymerase *rpoB*) and clinical isolates (cardiolipin biosynthesis *cls* or PG production *pgsA*)^35,46,67–69^. In fact, a few DAP^R^ strains with cross-reduced DAP/VCM susceptibility included in our study were found to carry additional mutations besides *mprF*. Apart from the proposed mechanism of the reduced DAP/VCM susceptibility, the current study deduced another possible pathway conferring reduced susceptibility to only DAP, which is not related to *mprF*. The single-DAP^R^ strain K-2 carrying only a *lacF* mutation showed an increased binding of cytochrome *c* compared to DAP^S^ strain, which was contradictory to DAP^R^ strains of cross-reduced DAP/VCM susceptibility group. This indicated a lack of correlation between increased surface positive charge and *lacF* mutation-associated DAP resistance, consistent with previous studies which demonstrated not all DAP^R^ isolates showed increment of surface positive charges^70,71^.

**Figure 3:**
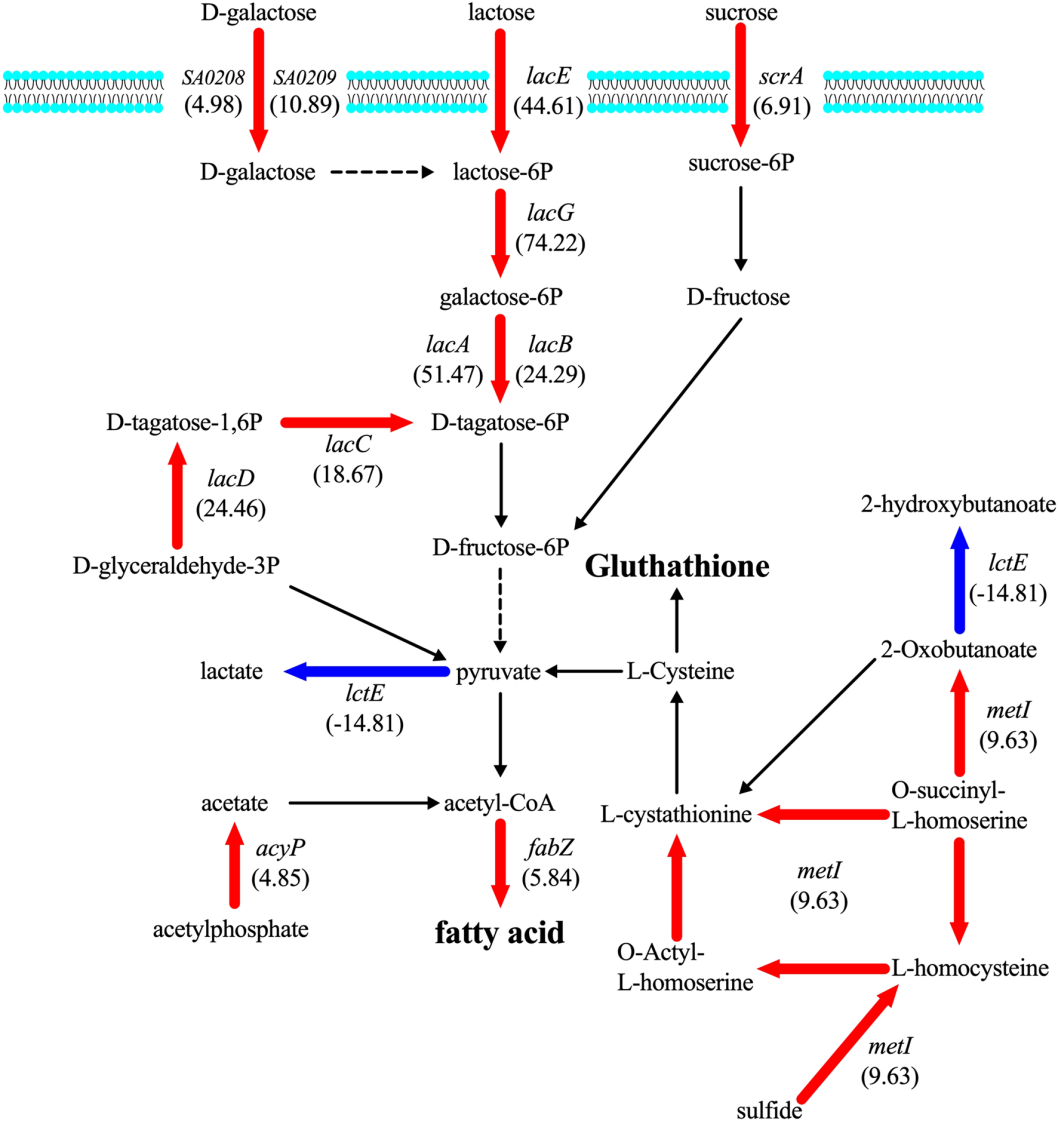
Gene expression in contribution to DAP resistance in DAP^R^ strain K-2. Acetyl-CoA, precursor for fatty acid metabolism, is produced via carbohydrate (lactose, tagatose, and sucrose) and cysteine methionine metabolism or acetate metabolism. The red arrows refer to gene upregulation. The blue arrows refer to gene downregulation.

DAP^R^ strain K-2 carrying mutations in *lacF*, a lactose phosphotransferase system (PTS), had reduced susceptibility to only DAP (Table 1). The association between mutations affecting carbohydrate transportation and resistance to cationic peptide (including DAP) has been demonstrated in *S*. *aureus*, *E. faecalis*, and *Listeria monocytogenes*^72–74^. *L. monocytogenes* carrying mutation in the mannose PTS system showed resistance to bacteriocins, one of the cationic peptides capable of making pore-like structures in the membrane just as DAP, due to lower glucose consumption rate^74^. A previous study also reported that the bactericidal effect of DAP is enhanced by increased glucose concentration that eventually induces lysis protein activity^75^. Thus, reduced DAP susceptibility in the K-2 strain carrying a *lacF* mutation seems to be a result of decreased cell lysis caused by lowered glucose consumption. In addition, DAP^R^ strain K-2 demonstrated increased expression of genes involved in cysteine and methionine metabolism, which generates GSH (Supplemental Table 6, Fig. 3). This compound is commonly known to have antioxidant activity, protecting prokaryotic and eukaryotic cells from oxidative stresses^76^. However, the exact regulatory pathway(s) of GSH in conferring reduced DAP susceptibility is not experimentally proven, although mutation in GSH has been reported to cause DAP resistance in *E. faecalis*^77^. Our results indicated that increased GSH metabolism coupled with reduced cell lysis are the possible cellular adaptations protecting bacteria with *lacF* mutations from DAP toxicity.

This study concluded that cross-reduced susceptibility of MRSA to DAP and VCM is associated with *mprF* mutations. The reduction of DAP and VCM susceptibility is mainly mediated by alteration of bacterial surface charge and increased L-PG production, while increased CW thickness is marginally involved. We also revealed a novel pathway leading to DAP resistance that is not related to *mprF*. Moreover, reduced DAP susceptibility without a parallel reduction of VCM susceptibility, as observed in our studied strain, is believed to be caused by alterations in cellular metabolisms ensued from *lacF* mutations, but the exact mechanisms remain to be elucidated.

## Materials and methods

### Bacterial strains and drug susceptibility testing

The bacterial isolates used in this study included 12 pairs of DAP^S^ and DAP^R^ strains, each collected from the same patient before and after DAP treatment (Supplemental Table 1), and 32 VISA strains isolated from patients receiving VCM therapy (Supplemental Table 2). All bacteria were kept in a final concentration of 40% glycerol at -80°C. Unless otherwise stated, the bacterial glycerol stocks were revived through cultivation in MH broth (Becton Dickinson, USA) at 37°C with constant agitation.

Two methods of drug susceptibility tests were employed in this study: Etest for determining DAP and VCM MIC for all studied strains, and broth microdilution for determining sensitivity toward DAP and VCM. Etests were performed following the guidelines of the Clinical and Laboratory Standard Institute. Briefly, each bacterial culture with 0.5 McFarland turbidity was streaked onto an MH agar plate, and DAP and VCM Etest strips (bioMérieux, France) were placed on the bacterial lawn. The inhibition zone break points for each isolate were read after 48 h. For the broth microdilution method, the ranges of DAP (0.5, 1, 1.5, 1.75, 2, 2.25, 2.5, and 3 mg/L) and VCM (0.5, 1, 1.5, 2, 2.25, 2.5, 3 and 4 mg/L) were tested against each bacterial culture. According to their susceptibility profile, each strain was classified as single DAP- or VCM-resistance or cross-reduced susceptibility to DAP and VCM.

### Growth curve and doubling time

Growth kinetics of bacterial strains in the DAP treatment group were determined. Overnight bacterial culture was adjusted to an optical density (OD) at 600 nm (OD_600_) of 0.3. Following that, the OD-adjusted culture was diluted 1:1000 with fresh brain–heart infusion broth (Becton Dickinson, USA), yielding a final concentration of 10^5^ colony forming unit (CFU)/mL. Bacterial suspensions were then incubated at 37°C with continuous agitation at 25 rpm in a temperature gradient rocking incubator (TVS126MB; ADVANTEC, Japan). The bacterial density at OD_600_ was recorded every 5 min over a period of 24 h. Growth curves were then generated by plotting OD measurements against time, and doubling time of bacteria was then determined with the equation described previously^7,78^.

### Population analysis profiling area under the curve (PAP-AUC) analysis

PAP-AUC is adapted from previous studies to confirm the DAP and VCM MICs of clinical MRSA isolates^7,79^. Briefly, an overnight culture of a MRSA strain was adjusted to an OD_600_ of 0.3 and serially diluted tenfold over a range of 10^−3^ to 10^−10^. Then, 100 µL of each dilution was spread on drug-free MH agar, MH agar with DAP (0.5, 0.75, 1, 1.5, 2, and 3 mg/L), and MH agar with VCM (0.5, 1, 2, 3, and 4 mg/L). After 48 h, the number of bacterial colonies was calculated and plotted semilogarithmically.

### DNA extraction and purification

Genomic DNAs of all studied strains were extracted from 20 mL of overnight culture grown in tryptic soy broth (Becton Dickinson, USA) by phenol–chloroform method and purified by DNeasy Blood and Tissue Kit (Qiagen, German) according to the manufacturer’s instructions. The DNA concentration was measured by a NanoDrop Lite spectrophotometer (Thermo Scientific, USA) and PicoGreen dsDNA assay kit (Invitrogen, USA).

### Multilocus sequence typing (MLST) and SNP determination by whole-genome sequencing

Each DAP^S^ strain was used as reference sequences for comparison. The integrative analysis of genomic DNA sequence was performed without size selection using Nextera Mate Pair Library Prep Kit following standard protocols and MiSeq instrument (2 × 301 bp) with the MiSeq reagent kit version 3 (Illumina, USA). Quality trimming was performed with FASTQ Toolkit version 2.0.0, and quality-trimmed sequences were assembled by the Velvet de novo assembly version 1.2.10 algorithm.

For DAP^R^ strains, sample libraries were prepared with the Nextera XT DNA Sample Preparation and Index Kits. The prepared DNA libraries were sequenced using MiSeq platform (Illumina, USA) with 300-bp paired end reads. The genetic backgrounds of clinical MRSA isolates were characterized by MLST, which involved determining the sequences of ~ 450-bp internal fragments of housekeeping genes (*arcC*, *aroE*, *glp*, *gmk*, *pta*, *tpi*, and *yqiL*) and compared with the reference genes on “Center for Genomic Epidemiology” website (https://cge.cbs.dtu.dk/services/MLST/). On the other hand, other gene mutations were identified by mapping the sequenced genomes against corresponding reference sequences by using CLC Genomics Workbench software (Qiagen, German). The sequence mapping satisfied with average coverage reads of over 40 across the whole reference genome. Genome sequences with coverage reads less than 10, or with equal or greater than 60% differences compared to that of reference (for those with coverage greater than 10) were called for analysis as potential variants (SNPs, deletion or insertion mutations). All the potential variants were verified by PCR and Sanger sequencing with an ABI3130 × 1 Genetic Analyzer (Applied Biosystems, USA).

### Gene replacement into the chromosome

To investigate the effect of the *mprF* mutation (L291I), identified in DAP^R^ isolates of each patient, on drug susceptibility, gene replacement was performed using the pKOR1 plasmid^30,80^. In brief, *mprF* genes were amplified from each H-1 (DAP^S^) and H-5 (DAP^R^) strain with primer sets listed in Supplemental Table 3. The PCR fragments were individually cloned into the pKOR1 plasmid using Gateway BP Clonase II enzyme mix (Thermo Scientific, USA), and recombinant plasmids were selected through CcdB-based positive selection system in *Escherichia coli* DH5α. The plasmid-carrying wild-type *mprF* gene was then introduced into DAP^R^ strain H-5, while the mutated *mprF* gene was transformed into DAP^S^ strain H-3. This was achieved by electroporation using NEPA21 electroporator (NEPAGENE, Japan) following the parameters reported previously^81^. Chromosomal gene replacement involved single-crossover plasmid integration at 43°C followed by overnight incubation in drug-free medium at 37°C to eliminate the plasmid. Anhydrotetracycline was used to select for non-plasmid-carrying mutants. The presence of gene mutations was confirmed by PCR and targeted gene sequencing with an ABI3130 × 1 Genetic Analyzer (Applied Biosystems, USA).

### In vitro induction by stepwise DAP exposure

Overnight bacterial cultures (C-1 and K-1 strains) were streaked onto MH agar supplemented with 50 mg/L calcium and a range of concentrations of DAP (0.5–4 mg/L). After incubation at 37°C for 2 days, the colonies grown on MH agar containing the highest concentration of DAP were picked and then streaked again onto fresh MH agar containing DAP at different concentrations. The colonies that could grow on 4 mg/L DAP were further investigated for their DAP and VCM MICs, along with the presence of *mprF* or *lacF* mutation by Sanger sequencing.

### Transmission electron microscopy (TEM)

CW thickness of all bacterial isolates from DAP treatment group were determined using TEM as previously described and visualized with TEM (Hitachi H-7600, Japan)^82,83^. Thirty cells of each bacterial strain were examined for CW thickness measurement at nearly equatorial cut surfaces. The results were presented as means $\pm$ standard deviations.

### Evaluation of membrane surface charge

Cytochrome *c* binding assays were performed as previously^84^ described to measure membrane surface charges of bacterial isolates from the DAP treatment group and transformed mutants carrying a wild-type or mutated *mprF* gene. The amount of unbound cytochrome *c* (Sigma-Aldrich, USA) was determined with spectrophotometry at OD_410_. Cytochrome *c* binding values in each DAP^R^ strain were determined from three independent studies with normalization to DAP^S^ strains of the same set.

### Determination of L-PG production

PL extraction from clinical MRSA isolates was adapted from the Bligh-Dyer procedure^85^. Briefly, the pelleted cells of overnight cultures of DAP^S^ / DAP^R^ clinical isolates and transformed mutants were adjusted to an OD_620_ of 20 and digested with a mixture of chloroform/methanol/water (1.75:3.5:1.4; v/v/v). Chloroform and 0.85% KCl weighing 1.75 mL and 1.6 mL, respectively, were added sequentially to the mixture. The extracted organic layers were concentrated by evaporation before separation with TLC (Silica-Gel 60-W-F254s, Merck, USA) in chloroform/methanol/water (65:25:4; v/v/v). Lysyl-phosphatidylglycerol was visualized by ninhydrin spray (FUJIFILM Wako Pure Chemicals, Japan), while total PLs were detected with molybdenum blue (Merck, USA). The relative amount of L-PG and total PLs in each sample were determined by ImageJ software (Wayne Rasband, USA). The L-PG levels relative to total PLs of DAP^R^ strains were calculated from three independent studies by comparing with DAP^S^ strains of the same set.

### RNA extraction and RNA expression analysis

Overnight bacterial cultures (H-3/H-5 and K-1/K-2) diluted 1:100 in 10 mL of MH broth were incubated at 37°C to an OD_600_ of 0.8. The bacterial pellet was harvested and resuspended with 6 mL of precooled T_10_E_10_ buffer (10 mM Tris–HCl, 10 mM EDTA; pH 8.0), followed by the addition of 10 mg/L lysostaphin (Sigma-Aldrich, USA) for complete bacterial lysis. Consequently, 7 mL of acidic-phenol saturated with 20 mM NaOAc (pH 4.8) (FUJIFILM Wako Pure Chemicals, Japan) and 600 µL of 3 M NaOAc (pH 4.8) were added. The mixture was subjected to three cycles of 20 min freezing at -80 °C and 5 min thawing at 65 °C. Bacterial RNA was then extracted by the phenol–chloroform method, followed by ethanol precipitation. The RNA pellet was dissolved with DNase I, recombinant, RNase-free (Roche, Germany) and purified by RNeasy Mini Kit Part 2 (Qiagen, German) before re-extraction with phenol–chloroform and ethanol precipitation. Finally, ribosomal RNAs in total RNA preparations were depleted using the Ribo-Zero rRNA Removal Kit (Illumina, USA). The extracted RNAs were first converted into complementary DNA (cDNA) and subsequently made into double-stranded DNA (dsDNA) by PrimeScript Double Strand cDNA Synthesis Kit (Takara, Japan). The generated dsDNAs were then used as templates for cDNA library preparation using Nextera XT DNA Library Prep Kit (Illumina, USA) as previously described. The fold change of RNA expression between the DAP^S^ and DAP^R^ strains was determined by CLC Genomics Workbench software.

### Statistical analysis

Student’s *t* test was employed for all statistical analyses.

### Ethics approval and consent to participate

Ethics approval and consent to participate were not required. All bacteria were isolated from hospitals in Japan as part of the standard patient care and used anonymously.

(To Editors: For consideration on this issue, ethics approval is not required following the ethical guidelines for medical and health research involving human subjects by Ministry of Health, Labour and Welfare, Japan since this study analyzed bacteria which were isolated as a clinical specimen and patients’ personal health information could not be accessed.

(https://www.mhlw.go.jp/stf/seisakunitsuite/bunya/hokabunya/kenkyujigyou/i-kenkyu/index.html).

## Supplementary information

Supplementary file1

Supplementary file2

## Data Availability

The genome sequence has been deposited at DDBJ/Genbank: PRJDB9008 (BioProject) DRA009427 (Raw data).
