# Supplementary material for: Association of mprF mutations with cross-resistance to daptomycin and vancomycin in methicillin-resistant Staphylococcus aureus (MRSA)
Source: Sci Rep. 2020 Sep 30;10:16107. doi: 10.1038/s41598-020-73108-x (PMC7527455; doi:10.1038/s41598-020-73108-x)

**Association of *mprF* mutations with cross-resistance to daptomycin and vancomycin in methicillin-resistant *Staphylococcus aureus* (MRSA)**

Kanate Thitiananpakorn^1^, Yoshifumi Aiba^1^, Xin-Ee Tan^1^, Shinya Watanabe^1^, Kotaro Kiga^1^, Yusuke Sato’o^1^, Tanit Boonsiri^1^, Feng-Yu Li^1^, Teppei Sasahara^1^, Yusuke Taki^1^,
Aa Haeruman Azam^1^, Yuancheng Zhang^1^, and Longzhu Cui^1^*

^1^ Division of Bacteriology, Department of Infection and Immunity, Faculty of Medicine, Jichi Medical University, 3311-1, Yakushiji, Shimotsuke-shi, Tochigi 329-0498, Japan.

* Corresponding author, E-mail address: longzhu@jichi.ac.jp

**Supplemental Figure 1:**

**
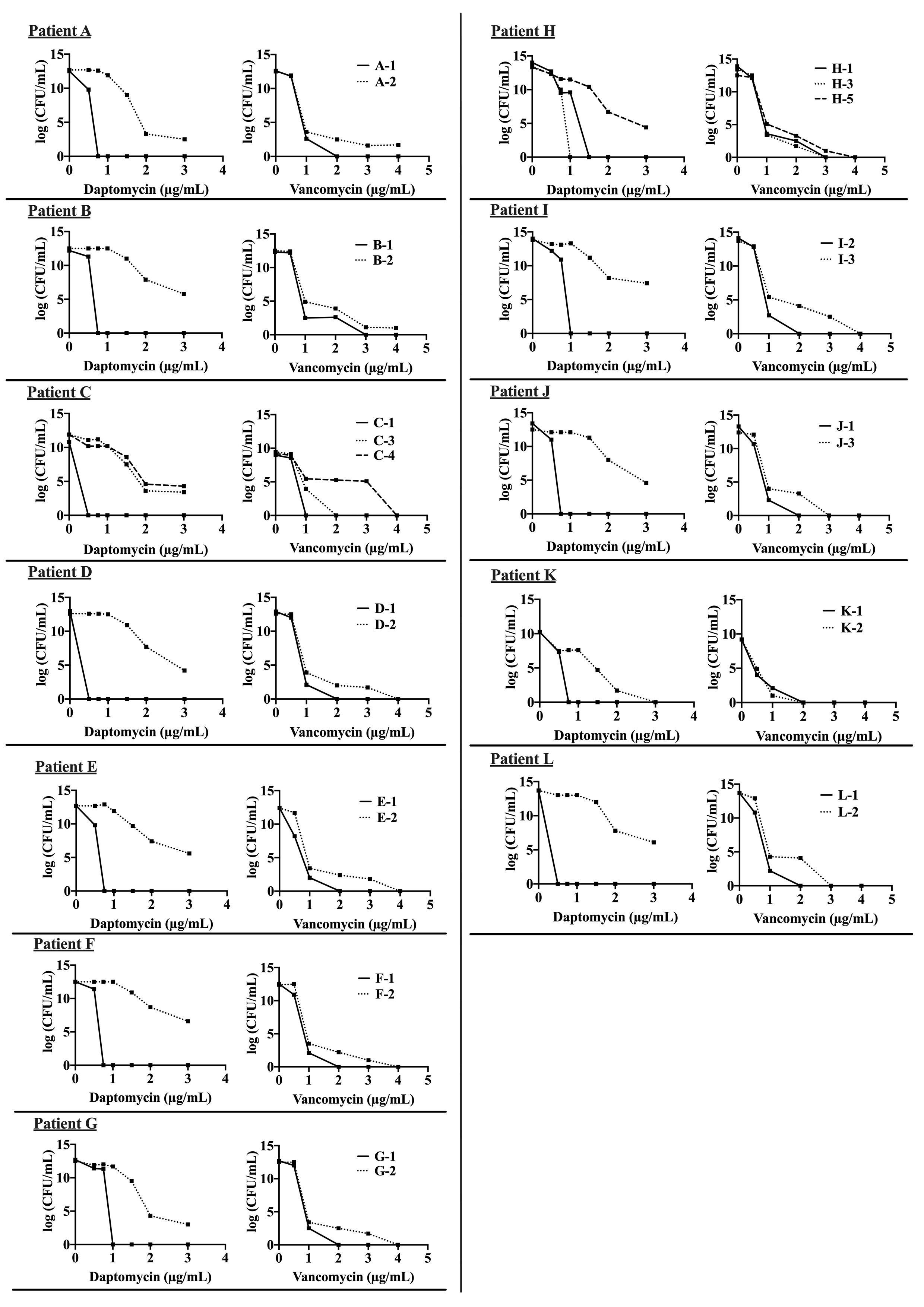
**

**Supplemental Figure 2:**


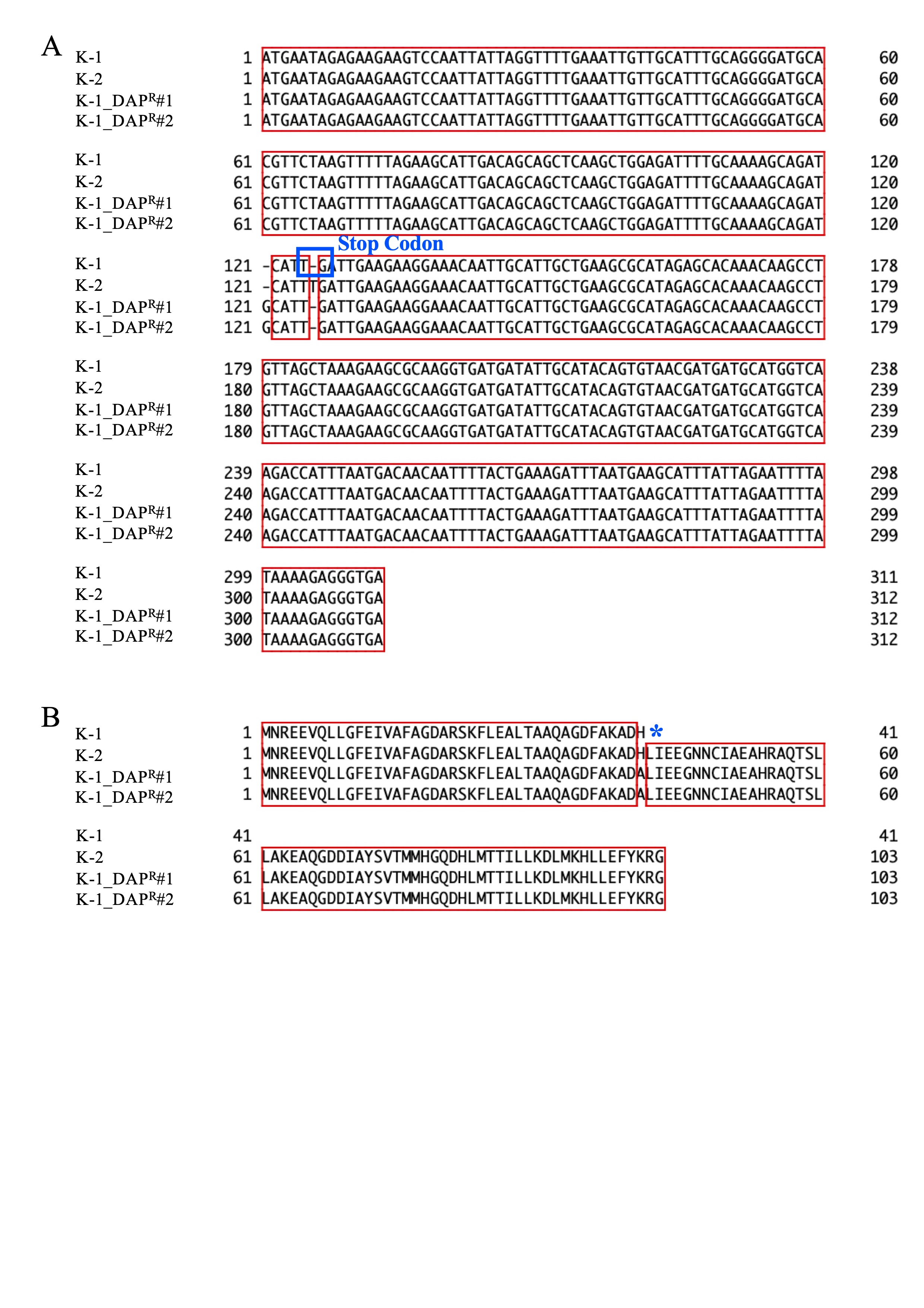

Supplement: Supplementary file 1 — Supplementary file1 [file 41598_2020_73108_MOESM1_ESM.docx]
