# Supplementary material for: Association of mprF mutations with cross-resistance to daptomycin and vancomycin in methicillin-resistant Staphylococcus aureus (MRSA)
Source: Sci Rep. 2020 Sep 30;10:16107. doi: 10.1038/s41598-020-73108-x (PMC7527455; doi:10.1038/s41598-020-73108-x)
Supplement: Supplementary file 2 — Supplementary file2 [file 41598_2020_73108_MOESM2_ESM.docx]

Association of *mprF* mutations with cross-resistance to daptomycin and vancomycin in methicillin-resistant *Staphylococcus aureus* (MRSA)

Kanate Thitiananpakorn^1^, Yoshifumi Aiba^1^, Xin-Ee Tan^1^, Shinya Watanabe^1^, Kotaro Kiga^1^, Yusuke Sato’o^1^, Tanit Boonsiri^1^, Feng-Yu Li^1^, Teppei Sasahara^1^, Yusuke Taki^1^,
Aa Haeruman Azam^1^, Yuancheng Zhang^1^, and Longzhu Cui^1^*

^1^ Division of Bacteriology, Department of Infection and Immunity, Faculty of Medicine, Jichi Medical University, 3311-1, Yakushiji, Shimotsuke-shi, Tochigi 329-0498, Japan.

* Corresponding author, E-mail address: longzhu@jichi.ac.jp

Supplemental Table 1. *Staphylococcus aureus* isolates from patients with DAP treatment

| Patient | Strain | Collected Source | DAP Exposure | DAP MIC from original source | | JMUB No.^a^ | Reference |
| --- | --- | --- | --- | --- | --- | --- | --- |
|  |  |  |  | DAP (mg/L) | Methods |  |  |
| A | A-1 | Wound | -^b^ | 0.25 | VITEK2 | JMUB480 | 36 |
|  | A-2 | Wound | 7 days | 4 | VITEK2 | JMUB481 | 36 |
| B | B-1 | Purulent | - | 0.5 | VITEK2 | JMUB483 | 36 |
|  | B-2 | Purulent | 14 days | 4 | VITEK2 | JMUB484 | 36 |
| C | C-1 | Purulent | - | 0.25 | VITEK2 | JMUB486 | 36 |
|  | C-2 | Purulent | 15 days | 0.25 | VITEK2 | JMUB487 | 36 |
|  | C-3 | Purulent | 26 days | 4 | VITEK2 | JMUB488 | 36 |
|  | C-4 | Purulent | 33 days | 4 | VITEK2 | JMUB489 | 36 |
| D | D-1 | Purulent | - | 0.25 | VITEK2 | JMUB491 | 36 |
|  | D-2 | Purulent | 24 days | 4 | VITEK2 | JMUB492 | 36 |
| E | E-1 | Blood | - | 0.5 | WalkAway40 puls | JMUB473 | This study |
|  | E-2 | Wound | 21 days | >1 | WalkAway40 puls | JMUB474 | This study |
| F | F-1 | Sputum | - | 0.5 | WalkAway40 puls | JMUB475 | This study |
|  | F-2 | Brain abscess | 45 days | >1 | WalkAway40 puls | JMUB476 | This study |
| G | G-1 | Wound | - | 0.5 | WalkAway40 puls | JMUB477 | This study |
|  | G-2 | Wound | 7 months | >1 | WalkAway40 puls | JMUB478 | This study |
| H | H-1 | N/A^c^ | - | ND | N/A | JMUB611 | This study |
|  | H-2 | N/A | 5 days | ND | N/A | JMUB612 | This study |
|  | H-3 | N/A | 27 days | ND | N/A | JMUB613 | This study |
|  | H-4 | N/A | 2 months | ND | N/A | JMUB614 | This study |
|  | H-5 | N/A | 3 months | ND | N/A | JMUB615 | This study |
| I | I-1 | Blood | - | 0.5 | BMD^d^ | JMUB1250 | This study |
|  | I-2 | Blood | - | 1 | BMD | JMUB1251 | This study |
|  | I-3 | Blood | 2 months | >2 | BMD | JMUB1252 | This study |
| J | J-1 | Blood | - | ND | N/A | JMUB1276 | This study |
|  | J-2 | Blood | 51 days | >1 | BMD | JMUB1277 | This study |
|  | J-3 | Blood | N/A | 2 | BMD | JMUB1278 | This study |
|  | J-4 | Blood | N/A | 2 | BMD | JMUB1279 | This study |
| K | K-1 | Blood | - | 0.5 | WalkAway40SI | JMUB1358 | This study |
|  | K-2 | Blood | 6 days | >1 | WalkAway40SI | JMUB1359 | This study |
| L | L-1 | Blood | - | 1 | WalkAway40SI | JMUB1360 | This study |
|  | L-2 | Blood | - | >1 | WalkAway40SI | JMUB1361 | This study |
|  | L-3 | Blood | 13 days | <0.5 | WalkAway40SI | JMUB1363 | This study |
| a) Jichi Medical University Bacterialbank number; b) Before daptomycin treatment; c) not available; d) broth microdilution method. | | | | | | | |

Supplemental Table 2. Vancomycin-intermediate *Staphylococcus aureus* (VISA) strains from patients with VCM treatment

| Strain Name | Year | Country | Code^a^ | MLST^b^ | Reference |
| --- | --- | --- | --- | --- | --- |
| NJ (HIP5836) | 1997 | USA | NRS4 | 5 | [7] |
| HIP07256 | 1999 | USA | NRS19 | 5 | [7] |
| LIM2 | 1995 | France | NRS36 | 247 | [7] |
| HIP09740 | 2000 | USA | NRS51 | 5 | [34] |
| HIP10540 | 2000 | USA | NRS73 | 8 | [34] |
| P1V44 | 1999 | Belgium | NRS272 | 247 | [34] |
| 99/3759-V | 1999 | UK | NRS39 | 247 | [7] |
| AMC11094 | 1997 | Korea | NRS49 | 5 | [7] |
| LY-1999-03 | 1998 | Oman | NRS65 | 372 | [34] |
| C2000001227 | 2000 | USA | NRS76 | 5 | [34] |
| NRS118 | 2002 | USA | NRS118 | 247 | [34] |
| NRS126 | 2000 | USA | NRS126 | 5 | [34] |
| 98141 | 1998 | France | N/A^c^ | 247 | [7] |
| MI (HIP5827) | 1997 | USA | NRS3 | 5 | [7] |
| SA MER | 1998 | France | NARSA | 5 | [34] |
| SA MER-S6 | 1998 | France | NRS12 | 5 | [34] |
| SA MER-S20 | 1998 | France | NRS14 | 5 | [34] |
| HIP06297 (PC) | 1998 | USA | NRS17 | 5 | [7] |
| HIP08926 | 2000 | USA | NRS23 | 5 | [34] |
| HIP09143 | 2000 | USA | NRS24 | 5 | [34] |
| HIP12864 | 2003 | USA | NRS402 | 5 | [34] |
| HIP13057 | 2004 | USA | NRS403 | 5 | [34] |
| HIP13036 | 2004 | USA | NRS404 | 5 | [34] |
| Mu50 | 1996 | Japan | NRS1 | 5 | [7] |
| HIP06854 | 1998 | USA | NRS18 | 5 | [34] |
| HIP09313 | 2000 | USA | NRS26 | 8 | [34] |
| HIP09662 | 2000 | USA | NRS28 | 8 | [34] |
| LY-1999-01 | 1998 | Oman | NRS63 | 372 | [34] |
| 99/3700-W | 1999 | UK | N/A | 239 | [7] |
| 28160 | 1998 | Africa | N/A | 247 | [7] |
| BR5 | 1998 | Brazil | NRS54 | 239 | [7] |
| a) NARSA's catalog number (NARSA: The Network on Antimicrobial Resistance in *Staphyloccocus aureus* (NARSA) (http://www.narsa.net); b) Multi-locus sequence typing; c) not available. | | | | | |

Supplemental Table 3. Primers and strains used in this study

| Target gene and region | Primer | Oligonucleotide sequence (5’-3’) |
| --- | --- | --- |
| Primer for sequence confirmation | | |
| *mprF* | mprF-F1 | CCA TAT TGT TCT GTT TGA GAT AG |
|  | mprF-F2 | GTT TTG TAG GAT TGT ACT GCA |
|  | mprF-F3 | CAA TTT TAT TAA TCA CAG TGG C |
|  | mprF-F4 | GAT CAA CAC ATG CCT TTA TAT C |
|  | mprF-R1 | CAA TAA AGG ACA TGA ATG ATA C |
|  | mprF-R2 | ACA GAT GTA TGC ATT TCA ATC |
|  | mprF-R3 | GCG CTT TAG ATA AGT ATT CTT C |
|  | mprF-R4 | CGA CTT AAC TTA AGC TCA TTT C |
| *agrA* | agrA-F | GCA GAA TTA AGA ACT CGA ATT |
|  | agrA-R | TGC ATT TGC TAG TTA TCT TGT |
| *lacF* | lacF-F | TAC ATT TAC TTA AGT GCT GGT G |
|  | lacF-R | CAC GTA TGC AAT TAA TAG AAA G |
| *hisF* | hisF-F | AGC TTA ATG CTA GAA GTG ATT G |
|  | hisF-R | GAT TAT CTT GTA AAA TTG CTG G |
| F1_0943 | 985260_F1-F | CAA AAA TTG ATG GAT GGT AC |
|  | 985260_F1-R | ACC TAG TCG TTC TAA AAC ATC T |
| 626772_G1 | 626772_G1-F | GTA AAA CAT AAA TCT CAA AGG C |
|  | 626772_G1-R | CCA TCT TCT GGA TAA CAA AT |
| L1_0548 | 611436_K3-F | CTT GTA TCA ACA TGG TAC ATT AG |
|  | 611436_K3-R | ACC TGT TCC TAA TAT CGC TAA |
| H1_0704 | 699807_H1-F | GCG TAC GAT TAT GTA TTT TTA AC |
|  | 699807_H1-R | TTA AAT GAC TTT TTG CAC CT |
| B1_1709 | 1840742^1840743_B1-F | TGT CTG AAT AAA TCG ATA AGG T |
|  | 1840742^1840743_B1-R | CGT TGA TCT ACG AAT CAT AAA |
| 255186_D1 | 255186_D1-F | GAT GAT TGT CCT TCA GCT TA |
|  | 255186_D1-R | AAA CAT CAT TTC TGT CTT ACC T |
| 472436_D1 | 472436_D1-F | TCC ATA GCC TTT GAA GAT AA |
|  | 472436_D1-R | AAT TCG GTA AAG GAT TTG T |
| Primer for gene substitution | | |
| M13M3 | pKOR1 plasmid | GTA AAA CGA CGG CCA GT |
| M13RV |  | CAG GAA ACA GCT ATG AC |
| Forward | pKOR1-*mprF* | GGGGACCACTTTGTACAAGAAAGCTGGGTAAATATTCTTATCTGTACCG |
| Reverse |  | GGGGACAAGTTTGTACAAAAAAGCAGGCTAACACTTAAGATTCATCCAC |
| *mprF*-extra | pKOR1-*mprF*-Chromosome | GTT AGG CGA CTT AAC TTA A |
|  |  |  |
| Strain | Description | |
| *S*. *aureus*-transformed strains | | |
| H-3_*mprF* (L291I) | allelic replacement with MprF (L291I) from H5 into H3-DS strain | |
| H-5_*mprF* (WT) | allelic replacement with wild type MprF from H1 into H5-DNS strain | |
| *E*. *coli* strain | | |
| DH5α | Gene amplification, Amp^S^ | |
| WT: wild type |  |  |

Supplemental Table 4. Daptomycin and vancomycin MICs determined by using broth microdilution method

| Patient | Strain | MICs (mg/L) | |
| --- | --- | --- | --- |
|  |  | DAP | VCM |
| Cross-reduced susceptibility group (to DAP and VCM) | | | |
| A | A-1 | 1 | 1 |
|  | A-2 | >3 | 2 |
| B | B-1 | 1 | 1 |
|  | B-2 | >3 | 1.5 |
| C | C-1 | 1 | 1.5 |
|  | C-3 | >3 | 2.25 |
|  | C-4 | >3 | 2.25 |
| D | D-1 | <0.5 | 1 |
|  | D-2 | 3 | 1.5 |
| E | E-1 | 1 | 1 |
|  | E-2 | >3 | 1.5 |
| F | F-1 | 1 | 1 |
|  | F-2 | >3 | 2 |
| G | G-1 | 0.5 | 1 |
|  | G-2 | 2.5 | 1.5 |
| H | H-1 | 1 | 2 |
|  | H-3 | 1 | 2 |
|  | H-5 | >3 | 2.25 |
|  | H-3 (*mprF*_H-5) | >3 | 3 |
|  | H-5 (*mprF*_H-1) | 1 | 2 |
| I | I-2 | 1 | 2 |
|  | I-3 | >3 | >4 |
| J | J-1 | 1 | 1.5 |
|  | J-3 | >3 | 2 |
| L | L-1 | 1 | 1.5 |
|  | L-2 | >3 | 4 |
| Reduced susceptibility to only DAP group | | |  |
| K | K-1 | 1 | 1.5 |
|  | K-2 | 2 | 1.5 |

Supplemental Table 5. Representatives of genes differentially expressed between DAP^R^ strain H-5 and DAP^S^ strain H-3

| N315 locus tag | Gene | Function | Fold change (H-5/H-3) |  |
| --- | --- | --- | --- | --- |
|  |  |  |  |  |
| Metabolism | |  |  |  |
| [C] Energy production and conversion | | | | |
| SA0910 | *qoxD* | Cytochrome aa3 quinol oxidase, subunit IV | 5.6 |  |
| SA1451 |  | Luciferase family oxidoreductase, group 1 | 4.3 |  |
| SA1988 |  | Zinc-binding alcohol dehydrogenase family protein | 4.0 |  |
| SA2184 | *narH* | Nitrate reductase, beta subunit | - 4.9 |  |
| [E] Amino acid transport and metabolism | | | | |
| SA1216 |  | Oligoendopeptidase F (Peptidase family M3) | 4.7 |  |
| SA1980 |  | Alanine racemase | 4.3 |  |
| SA0668 | *pabA* | Glutamine amidotransferase of anthranilate synthase or aminodeoxychorismate synthase | - 9.3 |  |
| SA2319 | *sdaAB* | L-serine dehydratase, iron-sulfur-dependent, beta subunit | - 4.8 |  |
| [F] Nucleotide transport and metabolism | | | | |
| SA1041 | *pyrR* | Hypoxanthine phosphoribosyltransferase | 4.1 |  |
| [G] Carbohydrate transport and metabolism | | | | |
| SA0236 | *gatA* | PTS system, fructose subfamily, IIA component | 5.2 |  |
| SA1599 | *tal* | Transaldolase/Fructose-6-phosphate aldolase | 8.4 |  |
| SA1656 | *hit* | Diadenosine tetraphosphate (Ap4A) hydrolase | 7.5 |  |
| SA2362 |  | Ergot alkaloid biosynthesis protein, AFUA_2G17970 family | 5.3 |  |
| SA2435 | *pmi* | Mannose-6-phosphate isomerase, class I | 4.8 |  |
| SA0903 |  | Acyltransferase family | - 4.6 |  |
| SA1758 | *sak* | Staphylokinase/Streptokinase family | - 6.1 |  |
| [H] Coenzyme transport and metabolism | | | | |
| SA1412 | *hemN* | Putative oxygen-independent coproporphyrinogen III oxidase | - 4.5 |  |
| SA2390 | *panD* | Aspartate 1-decarboxylase | - 4.5 |  |
| [I] Lipid transport and metabolism | | | | |
| SA0223 | *fadA* | Acetyl-CoA C-acyltransferase | 4.3 |  |
| [P] Inorganic ion transport and metabolism | | | | |
| SA0333 | *fepC* | FTR1 family protein (Iron permease FTR1 family) | 4.0 |  |
| SA1977 | *htsC* | Proposed F420-0 ABC transporter, permease protein | 4.1 |  |
| SA0951 | *potB* | Putative 2-aminoethylphosphonate ABC transporter | - 5.1 |  |
| SA0110 | *sirB* | Proposed F420-0 ABC transporter, permease protein | - 11.9 |  |
| SA1212 | *opp-2D* | Nickel import ATP-binding protein NikD | - 6.0 |  |
| SA1219 | *pstA* | Phosphate ABC transporter, permease protein PstA | - 4.0 |  |
| [Q] Secondary metabolites biosynthesis, transport and catabolism | | | | |
| SA1419 |  | Malonyl-acyl carrier protein O-methyltransferase BioC (Methyltransferase domain) | 6.1 |  |
| SA0181 |  | Pyrimidine utilization protein B | - 4.2 |  |
| SA0301 | *psuG* | CRISPR-associated endonuclease Cas1 | - 5.1 |  |
| Information storage and processing | | | | |
| [J] Translation, ribosomal structure and biogenesis | | | | |
| SA1067 | *rpmB* | Ribosomal protein bL28 | 4.0 |  |
| SA1116 | *rpsO* | Ribosomal protein uS15 | 5.6 |  |
| SA1459 | *dtd* | D-tyrosyl-tRNA(Tyr) deacylase | 5.8 |  |
| SA1918 |  | tRNA threonylcarbamoyl adenosine modification protein | 5.6 |  |
| [K] Transcription | | | | |
| SA1191 | *glcT* | GTP cyclohydrolase II (PRD domain) | 11.1 |  |
| SA1351 | *argR* | arginine repressor | 7.9 |  |
| SA1851 | *rex* | Transcriptional regulator | 6.0 |  |
| SA1998 | *lacR* | CRISPR locus-related DNA-binding protein | 4.0 |  |
| SA2089 | *sarR* | Staphylococcal accessory regulator family (MarR family) | 4.0 |  |
| SA0559 |  | Ribosomal-protein-alanine acetyltransferase | - 6.0 |  |
| [L] Replication, recombination and repair | | | | |
| SA0987 | *rnhC* | Ribonuclease HIII | 5.0 |  |
| SA1180 | *sbcD* | Exonuclease SbcCD, D subunit (DNA repair) | 4.7 |  |
| SA1305 | *hup* | Integration host factor, beta subunit | 4.9 |  |
| SA1489 | *tag* | DNA-3-methyladenine glycosylase I | 4.6 |  |
| Cellular process and signaling | | | | |
| [M] Cell wall / membrane / envelop biogenesis | | | | |
| SA2291 | *fnbA* | LPXTG cell wall anchor domain | - 6.0 |  |
| [O] Post-translational modification, protein turnover and chaperones | | | | |
| SA1179 |  | Multicomponent Na+:H+ antiporter | - 6.0 |  |
| SA1627 | *splF* | Peptidase Do (Trypsin-like peptidase domain) | - 4.1 |  |
| SA1629 | *splC* | Periplasmic serine peptidase DegS | - 4.0 |  |
| SA1631 | *splA* | Periplasmic serine peptidase DegS | - 6.1 |  |
| [T] Signal transduction mechanisms | | | | |
| SA2153 |  | LytTr DNA-binding domain | 4.2 |  |
| [U] Intracellular trafficking, secretion and vesicular transport | | | | |
| SA0825 | *spsA* | Signal peptidase I | 4.3 |  |
| Poorly characterized | | | | |
| [S] Function unknown | | | | |
| SA0275 | *essB* | type VII secretion protein EssB | 6.0 |  |
| SA0355 |  | Abi-like protein (CAAX protease) | 4.3 |  |
| SA0513 |  | HAD hydrolase, TIGR02253 family | 4.6 |  |
| SA0544 | *hemQ* | Chlorite dismutase (heme-binding protein) | 11.3 |  |
| SA0649 |  | Domain of unknown function (DUF296) | 5.6 |  |
| SA0664 |  |  | 4.8 |  |
| SA0772 |  | CsbD family protein | 5.7 |  |
| SA0914 |  | Domain of unknown function (DUF5011) | 7.9 |  |
| SA1167 |  | Cof-like hydrolase (haloacid dehalogenase-like hydrolase) | 7.1 |  |
| SA1187 | *plsY* | acyl-phosphate glycerol 3-phosphate acyltransferase | 4.6 |  |
| SA1752 | *hlb-1* | ß-hemolysin | 5.2 |  |
| SA1811 | *hlb-2* | a phospholipase C toward sphingomyelins | 113.8 |  |
| SA2093 | *ssaA* | CHAP domain-containing protein | 5.9 |  |
| SA2101 |  | Protein of unknown function (DUF1641) | 4.2 |  |
| SA2297 | *relP* | RelA/SpoT family protein | 5.8 |  |
| SA2443 | *asp3* | Accessory Sec system protein Asp3 | 4.3 |  |
| SA2447 | *sasA* | Signal peptidase I | 4.3 |  |
| SA0091 | *plc* | Phosphatidylinositol-specific phospholipase C, X domain | - 6.0 |  |
| SA0753 |  | L-lysine exporter | - 4.5 |  |
| SA0798 |  | Disulfide oxidoreductase | - 6.1 |  |
| SA0830 |  | MSEP-CTERM protein | - 4.6 |  |
| SA1209 |  | Cof-like hydrolase (haloacid dehalogenase-like hydrolase) | - 4.1 |  |
| SA1497 | *engB* | Ribosome biogenesis GTP-binding protein YsxC | - 4.6 |  |
| SA1613 |  | Putative membrane protein insertion efficiency factor | - 4.5 |  |
| SA1756 |  | Amidase | - 9.1 |  |
| SA1784 |  | dUTPase | - 6.0 |  |
| SA2163 |  | Protein of unknown function (DUF2871) | - 6.0 |  |
| SA2225 |  | Putative phosphoesterase (Calcineurin-like phosphoesterase) | - 9.1 |  |
| SA2450 |  | JNK-interacting protein leucine zipper II | - 6.1 |  |
| Unknown | | | | |
| SA0279 | *esaE* | Domain of unknown function (DUF5081) | 5.9 |  |
| SA0743 | *vwb* | Coagulase | 4.6 |  |
| SA0790 | *nagD* | Predicted sugar phosphatases of the HAD superfamily | 6.6 |  |
| SA0806 |  | U-34 5-methylaminomethyl-2-thiouridine biosynthesis protein | 4.0 |  |
| SA1161 |  | Cell division protein FtsL | 4.3 |  |
| SA1304 |  | Heptaprenyl diphosphate synthase (HEPPP synthase) subunit 1 | 4.4 |  |
| SA2118 |  | Protein of unknown function (DUF3397) | 4.0 |  |
| SA2139 |  | Formate dehydrogenase, alpha subunit | 4.3 |  |
| SA2331 |  | YozE SAM-like fold | 5.3 |  |
| SA2491 |  | Short repeat of unknown function (DUF308) | 4.5 |  |
| SAS066 | *agrD* | Cyclic lactone autoinducer peptide | 6.6 |  |
| SA0047 |  | Phenylacetic acid degradation protein paaN | - 15.3 |  |
| SA0203 |  | Staphylococcus tandem lipoproteins (Csa1 family) | - 4.5 |  |
| SA0382 | *ssl1* | Exotoxin | - 4.6 |  |
| SA1791 |  | DnaD domain protein | - 4.6 |  |
| SA2000 |  | Conserved hypothetical protein | - 4.5 |  |
| Mobile and extrachromosomal element functions | | | | |
|  |  | Terminase large subunit | 8.1 |  |
|  |  | Conserved phage-associated protein | 7.2 |  |
|  |  | Phage major capsid protein, HK97 | 11.8 |  |
|  | *ear* | Putative phage terminase, small subunit, P27 family | - 4.7 |  |
|  |  | Phi ETA orf 18-like protein | - 4.5 |  |
|  |  | Phage tail tape measure protein, TP901 family, core region | - 4.1 |  |
|  |  | Oxidoreductase (Phage antirepressor protein KilAC domain) | - 4.6 |  |
|  |  | Phage DNA-binding protein | - 6.6 |  |
|  |  | Phage protein | - 6.9 |  |

Supplemental Table 6. Representatives of genes differentially expressed between DAP^R^ strain K-2 and DAP^S^ strain K-1

| N315 locus tag | Gene | Function | Fold change (K-2/K-1) |  |
| --- | --- | --- | --- | --- |
|  |  |  |  |  |
| Metabolism |  |  |  |  |
| [C] Energy production and conversion | | | | |
| SA1236 | *acyP* | Carbamoyltransferase HypF | 4.8 |  |
| SA0232 | *lctE* | L-lactate dehydrogenase | -14.8 |  |
| [E] Amino acid transport and metabolism | | | | |
| SA0347 | *metI* | Cystathionine beta-lyases/cystathionine gamma-synthases | 9.6 |  |
| SA1239 | *brnQ3* | Component of the transport system | 4.2 |  |
| SA2081 |  | Urea transporter | 8.8 |  |
| [G] Carbohydrate transport and metabolism | | | | |
| SA0208 | *malF* | Putative 2-aminoethylphosphonate ABC transporter | 5.0 |  |
| SA0209 |  | Maltose ABC transporter, permease protein | 10.9 |  |
| SA0318 | *ulaA* | PTS ascorbate transporter subunit IIC | 4.3 |  |
| SA1991 | *lacG* | 6-phospho-beta-galactosidase | 74.2 |  |
| SA1992 | *lacE* | PTS system, lactose-specific IIC component | 44.6 |  |
| SA1994 | *lacD* | tagatose 1,6-diphosphate aldolase | 24.5 |  |
| SA1995 | *lacC* | tagatose-6-phosphate kinase | 18.7 |  |
| SA1996 | *lacB* | D-galactose 6-phosphate degradation | 24.3 |  |
| SA1997 | *lacA* | D-galactose 6-phosphate degradation | 51.5 |  |
| SA2167 | *scrA* | PTS system lactose-specific IIBC component | 6.9 |  |
| SAS020 |  | catalytic activity (Phosphoglycerate mutase family protein) | 4.2 |  |
| [H] Coenzyme transport and metabolism | | | | |
| SA2215 | *bioD* | Dethiobiotin synthase | - 5.1 |  |
| [I] Lipid transport and metabolism | | | | |
| SA1901 | *fabZ* | beta-hydroxyacyl-(acyl-carrier-protein) dehydratase FabZ | 5.8 |  |
| [P] Inorganic ion transport and metabolism | | | | |
| SA0417 |  | Neurotransmitter:sodium symporter activity | 5.8 |  |
| Information storage and processing | | | | |
| [J] Translation, ribosomal structure and biogenesis | | | | |
| SA0009 | *serS* | Serine-tRNA ligase | 4.4 |  |
| SA0652 |  | Cys-tRNA(Pro) deacylase | 5.8 |  |
|  | | | | |
| [K] Transcription | | | | |
| SA1665 |  | Cro/Cl family transcriptional regulator | - 5.2 |  |
| [L] Replication, recombination and repair | | | | |
| SA0538 | *ung* | Uracil-DNA glycosylase | - 4.5 |  |
| [M] Cell wall / membrane / envelop biogenesis | | | | |
| SA0205 |  | M23/M37 peptidase domain-containing protein | 4.8 |  |
| [O] Post-translational modification, protein turnover and chaperones | | | | |
| SA1121 |  | Predicted Zn-dependent peptidases | 4.2 |  |
| SA1543 |  | Peroxiredoxin | 5.8 |  |
| SA2370 |  | Putative flavoprotein monooxygenase | 8.6 |  |
| SA2414 | *gpxA2* | Putative glutathione peroxidase Gpx7 | 6.8 |  |
|  |  | Protease subunit of ATP-dependent Clp proteases | 7.7 |  |
| [V] Defense mechanism | | | | |
| SA0135 | *phnE1* | Phosphonate ABC transporter | - 9.3 |  |
| SA0851 | *opp-4D* | Oligopeptide ABC transporter | - 4.1 |  |
| SA1156 |  | ABC transporter, ATP-binding protein | 4.8 |  |
| Poorly characterized | | | | |
| [R] General function prediction only | | | | |
| SA0683 | *queF* | NADPH-dependent 7-cyano-7-deazaguanine reductase | 5.8 |  |
| [S] Function unknown | | | | |
| SA1136 |  | energy coupling factor transporter S component ThiW | 4.4 |  |
| SA2301 |  | DedA family protein | 4.9 |  |
| SA1292 |  | MazG nucleotide pyrophosphohydrolase | - 4.1 |  |
| Unknown | | | | |
| SA0211 |  | Inositol 2-dehydrogenase | 5.9 |  |
| SA0400 | *lpl4* | Staphylococcus tandem lipoproteins | 4.8 |  |
| SA0403 | *lpl3* | Staphylococcus tandem lipoproteins | 5.8 |  |
| SA0489 | *mrnC* | Correct processing of 23S rRNA precursor | 7.8 |  |
| SA0561 |  | Putative cytosolic protein (YwhD) | 6.8 |  |
| SA0710 |  | Probable autolysin LDP | - 7.3 |  |
| SA1317 |  | Uncharacterized Protein | - 4.6 |  |
| SA2372 |  | Uncharacterized protein | - 4.1 |  |
| SA2454 |  | Acetyltransferase (GNAT) domain | - 4.1 |  |
| Mobile and extrachromosomal element functions | | | | |
| SA0195 |  | Phage holin | 6.3 |  |
|  |  | Phage-like protein | 5.1 |  |
| SA1793 |  | beta-lactamase superfamily domain protein (Phage) | - 4.1 |  |
|  |  | Phage | - 7.3 |  |
|  |  | Phage packaging | - 6.2 |  |
